# AE-HGNN: attention-enhanced hypergraph neural networks for interpretable stress prediction through higher-order dependency modeling

**DOI:** 10.3389/frai.2026.1844784

**Published:** 2026-07-07

**Authors:** Bindu Garg, Manisha Kasar, Renuka Mane, Massimo Donelli, Achin Jain, Arun Kumar Dubey

**Affiliations:** 1 Department of Computer Science and Engineering, Bharati Vidyapeeth (Deemed to be University) College of Engineering, Pune, India; 2 School of Computer Engineering and Technology, Dr. Vishwanath Karad MIT World Peace University, Pune, India; 3 Department of Civil, Environmental and Mechanical Engineering, University of Trento, Trento, Italy; 4 Department of Information Technology, Bharati Vidyapeeth's College of Engineering, New Delhi, India

**Keywords:** attention mechanism, higher-order dependencies, hypergraph neural networks, non-invasive monitoring, stress prediction

## Abstract

Stress is a significant contributor to the deterioration of mental and physical health, including conditions such as anxiety, depression, and cardiovascular issues. Traditional machine learning techniques like SVM, Decision Trees, and DNNs have been used for stress prediction using physiological and behavioral data, but they struggle to capture complex, high-order interactions among stress indicators and require extensive preprocessing of noisy signals. To address these limitations, a novel Stress Prediction System based on Attention Enhanced Hypergraph Neural Networks (AE-HGNN) is proposed that models higher-order relationships among environmental and behavioral factors such as humidity, temperature, and step count. The proposed attention mechanism adds attention weights to each factor; unlike conventional graph-based models limited to pairwise relationships, the attention enhanced hypergraph neural network leverages hyperedges to represent multi-node dependencies, significantly improving classification performance and robustness. The primary contributions of this work involve the design of an Attention Enhanced-HGNN architecture for stress classification that models higher-order dependencies by assigning attention weights to each factor. The proposed model achieves a test accuracy of 99.75% (5-fold cross-validated mean: 98.44% ± 0.56%) with optimized hyperparameters including a learning rate of 0.01, a hidden dimension of 128, and an epoch size of 150. This performance significantly outperforms existing methods such as Random Forest (87.3%), SVM (82.8%), and standard DNNs (90.7%), confirming the model's potential for non-invasive, real-time stress monitoring via wearable technologies and mobile health platforms.

## 1 Introduction

Stress is a natural response of a person physiologically and psychologically when subjected to physical or emotional factors that contribute to an individual's well-being and performance. Factors like workload, financial instability, social interactions, and environmental conditions have all been considered as factors that arise to this condition (Chu et al., 2024). Short bursts of stress help in alertness and performance, but it deteriorates into severe health issues such as anxiety, depression, cardiac diseases, and weakened immune function (Chu et al., 2024). Corrective stress predictions are imperative for early intervention and effective mental health management, and these are measures that if taken timely can alleviate will effects (Akmandor and Jha, 2017). Identifying stress patterns early on can give people and health care providers an opportunity to deal with stressors before they become more severe health problems (Colizzi et al., 2020). The traditional methods used for stress prediction depend on the use of physiological signals as well as Logistic Regression, Support Vector Machines (SVM), Random Forest, Naïve Bayes, K-Nearest Neighbors, Decision Trees, and Deep Neural Networks (DNN), Artificial Neural Network (ANN) (Arya et al., 2024; Haque et al., 2023; Suryawanshi and Vanjale, 2023). However, in some scenarios they have proven promising, but they also have serious limitations. For example, they typically fail to identify complex, high dimensional relationships between stress related factors resulting in low generalization in real world applications (Haque et al., 2023; Li and Liu, 2020). In addition, these methods suffer from severe limitations in their capability to approximate complex interactions between several stress indicators, especially environmental and behavioral factors, necessary for reliable forecasting (Haque et al., 2023). Although these techniques are valuable, they often do not have interpretability and are often not able to provide good performance across different datasets (Arya et al., 2024; Li and Liu, 2020). For instance, physiological signals, as HRV and EDA, are loud but those two give important information, but such signals yet noisy and very long preprocessing is needed to use it in the predictive models (Haque et al., 2023). To address the need to model more complex interactions, the Hypergraph Neural Network (HGNN) was introduced to capture higher-order relationships among multiple data points (Lei et al., 2024; Ahmed et al., 2022). However, a major limitation of the standard HGNN architecture is its inability to differentiate the importance of various input features, treating all signals as equally relevant (Su et al., 2025). This can limit performance and, crucially, makes the model an uninterpretable “black box.” To solve these problems, an Attention-Enhanced Hypergraph Neural Network (AE-HGNN) is proposed. The primary contribution is a:

- The integration of a feature attention mechanism that enables the model to learn the relative importance of each input signal (humidity, temperature, and step count).
- Improving the model's interpretability, an attention mechanism was incorporated into the hypergraph convolution to dynamically learn the relative importance of behavioral and environmental features (temperature, humidity, and step count).
- Enhancing the robustness of stress classification by optimizing input representation through correlation analysis and data preprocessing.
- The proposed model achieves state-of-the-art accuracy (99.75%) with minimal misclassification, according to a thorough comparative study with GNNs and traditional ML models (SVM, Random Forest, DNN).

In order to predict stress, this work presents an Attention-Enhanced Hypergraph Neural Network (AE-HGNN), which uses hyperedges and attention weights to model higher-order dependencies between behavioral and environmental factors (temperature, step count, and humidity). The suggested model works on an optimized set of hyper parameters (learning rate = 0.01, hidden dimension = 128, epoch size = 150, dropout rate = 0.5) and incorporates a novel feature attention mechanism for interpretability and achieves a state-of-the-art accuracy (99.75%), enabling robust, non-invasive, and real-time stress monitoring, in contrast to traditional ML and GNN approaches that are restricted to pairwise interactions the proposed model offers a richer representation of multi modal physiological-environmental data while simultaneously enhancing novelty through its attention mechanism. The applications of the proposed Attention Enhanced-HGNN-based system include healthcare, workplace stress monitoring, mental health tracking, as well as interfacing with wearable devices. The system can be used in healthcare to monitor chronically ill patients to give out early warnings of complications associated with stress (Jafleh et al., 2024; Deng et al., 2023). Another way in which it can contribute to the workplace is facilitating organizations in identifying employees at risk of burnout and implementing targeted interventions (Jafleh et al., 2024; Li C. et al., 2024). The system can be integrated into mobile apps for the purpose of real time stress monitoring and personalized recommendations for mental health tracking (Li C. et al., 2024; Shajari et al., 2023). In addition, such wearable devices can provide a method of continuous stress monitoring to provide users with the ability to better manage their stress levels (Deng et al., 2023; Shajari et al., 2023). This system can transform stress related interventions and enhance overall quality of life by enabling early stress detection and management in an efficient and accurate way (Jafleh et al., 2024; Deng et al., 2023; Suryawanshi et al., 2022). This is the structure of the remainder of the paper. In Section 2, prior studies and current literature are thoroughly reviewed. In Section 3, the materials and approach are described. Results and experimental setup are described in Section 4. Comparative analysis, restrictions, and debate are the main topics of Section 5. Section 6 concludes the directions for future research.

The key contributions of this work are as follows:

- Design and implementation of an Attention-Enhanced Hypergraph Neural Network (AE-HGNN) architecture for stress classification using non-invasive environmental and behavioral data.
- Modeling of higher-order dependencies through hyperedges that capture complex, multi-node interactions beyond pairwise relationships in conventional GNNs.
- Integration of a feature-level and hyperedge-level attention mechanism to dynamically assign importance weights, enhancing both predictive performance and interpretability.
- Achievement of state-of-the-art accuracy of 99.75% with optimized hyperparameters, significantly outperforming traditional machine learning and deep learning baselines.

The remainder of this paper is organized as follows. Section 2 reviews related work on stress detection and graph-based neural networks in healthcare. Section 3 describes the dataset, preprocessing, hypergraph construction, and the proposed AE-HGNN architecture. Section 4 presents experimental results including performance metrics, confusion matrices, and attention-based interpretability. Section 5 discusses comparative analysis and statistical significance. Finally, Section 6 concludes the paper and outlines future research directions.

## 2 Related work

### 2.1 Evolution of stress monitoring

Automated stress detection has transitioned from invasive monitoring techniques toward less-intrusive computational models. Approximately 73% of stress detection studies utilize wearable sensors, such as ECG and GSR (Taskasaplidis et al., 2024). However, these high-accuracy methods are often costly, uncomfortable, and not easily accessible, which limits their widespread adoption. More specifically, sensor-based stress detection is challenged by real-time adaptation because physiological signals fluctuate due to environmental factors not associated with mental stress (Gedam and Paul, 2021). To mitigate these issues, recent research has begun exploring data augmentation and synthetic noise reduction to improve model generalization across diverse populations.

### 2.2 Non-invasive stress monitoring

Recent work on non-invasive stress detection has explored physiological signals (EEG, EDA/GSR, and HRV) and ubiquitous sensing (smartphones, wearables, and social-media traces) combined with machine learning to infer stress and related mental-health states (Gedam and Paul, 2021; Mohr et al., 2017). These studies show that HRV-derived features and multimodal fusion can improve detection performance, but often rely on small or controlled datasets [e.g., the HRV clustering study with 14 participants (Messaoud and Thamsuwan, 2025)], limiting generalizability. Practical deployment also faces engineering constraints such as sensor noise, battery life, user comfort, and privacy concerns; these remain active research directions (Rachakonda et al., 2018).

### 2.3 Graph neural networks in healthcare

Graph Neural Networks (GNNs) have been applied in healthcare to capture complex relationships in medical data, including disease prediction, patient similarity analysis, and neuroimaging applications (Paul et al., 2024; Sunil et al., 2024). GNNs are attractive because they model pairwise interactions and relational structure that standard architectures may miss, but they also raise challenges in scalability, interpretability, and privacy in clinical deployments (Zhao et al., 2024). For example, hybrid systems combining deep vision and sequential models (CNN–LSTM) have been proposed for real-time anomaly detection under federated learning settings; such systems achieve strong detection rates but demand substantial compute and communication resources and may suffer slower convergence due to distributed training (Nagamani and Kumar, 2024). We summarize representative GNN applications and their limitations in healthcare, emphasizing the need for models that capture higher-order relationships while remaining computationally practical.

### 2.4 Hypergraph neural networks

Hypergraph Neural Networks (HGNNs) extend GNNs by modeling higher-order relations (hyperedges) among multiple nodes simultaneously, which can better capture group-level or multimodal interactions (Hao et al., 2024). Recent works apply HGNNs to multimodal mental-health tasks (e.g., hypergraph models for depression recognition) and social recommendation problems by capturing co-occurrence and higher-order affinity patterns (Li X. et al., 2024; Khan et al., 2025; Zhang et al., 2025). While HGNNs often show improved representational power and predictive performance, they can be computationally more expensive and require careful hyperedge construction; robustness and interpretability remain important open issues.

Table 1 summarizes representative methods for stress prediction and highlights common limitations: reliance on wearable sensors, focus on pairwise relationships, limited adaptation to real-world variability, and computational/resource constraints. Our AE-HGNN addresses some of these limitations by modeling higher-order dependencies via hyperedges and by providing attention-derived attribution that we validate using model-agnostic methods (SHAP, LIME, and permutation importance) to improve interpretability and trustworthiness of the predictions.

**Table 1:** Comparison of earlier proposed stress prediction methods.

Model	Advantages	Disadvantages	Accuracy	Precision	Recall	F1-score
SVM, RF, NB etc. (Arya et al., 2024)	Different stress factors are considered, and multiple classifiers are compared	Training multiple models increases computational cost	89.54%	88.18%	90.14%	87.41%
Gradient boosting (Messaoud and Thamsuwan, 2025)	High accuracy on HRV-derived features	Small sample size (14 participants) limits generalizability	78.93%	86.68%	58.73%	57.79%
LDA (Schmidt et al., 2018)	Uses the WESAD multimodal dataset for wearable stress detection	Dataset limitations in subject diversity; reduced generalizability	93.12%	N/A	N/A	73.51%
MLP, DNN (Li and Liu, 2020)	Deep models enable rapid and accurate detection with multimodal inputs	Limited reliability when trained on small datasets	92.83%	N/A	N/A	91.07%
MLP (Dalmeida and Masala, 2021)	HRV-derived features from wearables provide useful signals	Limited hyperparameter exploration	83.33%	N/A	0.80 ± 0.06	0.72 ± 0.02
LSTM (Coutts et al., 2020)	Uses HRV data from 652 participants	Model accuracy requires further improvement	85%	N/A	N/A	N/A
Neural network (Patlar Akbulut et al., 2020)	High accuracy with multimodal physiological inputs	Requires expensive/complex sensors	92%	N/A	N/A	N/A

## 3 Materials and methods

**Figure 1:**
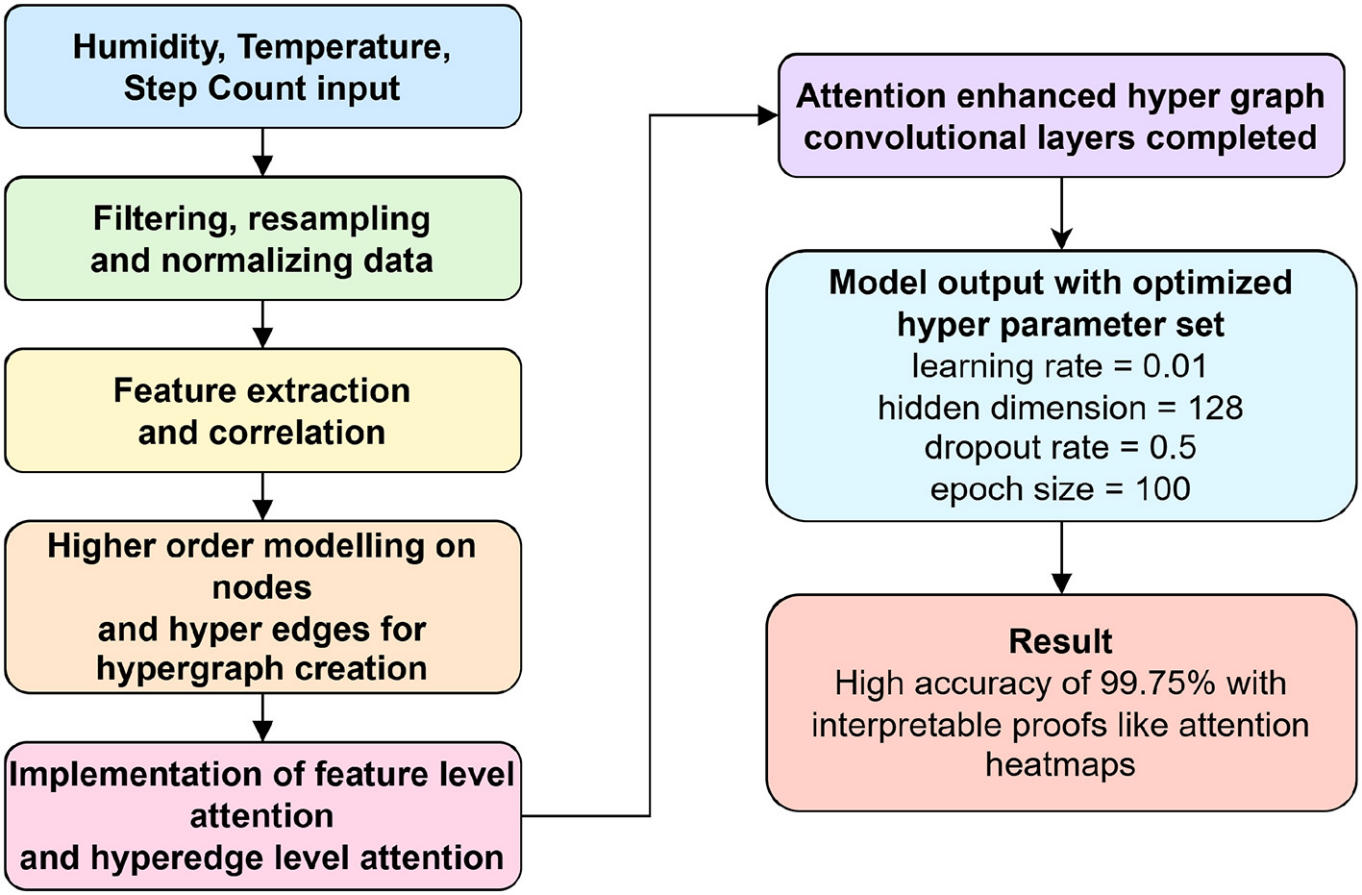
Flowchart of proposed work.

Attention Enhanced Hypergraph Neural Network (AE-HGNN) is proposed for modeling higher-order relationships by utilizing hyperedges to link multi-node dependencies unlike Graph Neural Networks (GNNs) (Nandan et al., 2025) that operate with second-order relationships only which is shown in Figure 1 The proposed attention enhanced hypergraph construction enables stress level classification by connecting people who share either humidity or temperature or step count trends.

### 3.1 Dataset and data preprocessing

In this study, the Stress-Lysis dataset is utilized, comprising environmental and behavioral data collected to analyze human stress levels. The dataset consists of 2,001 samples, providing a sufficient foundation for deep learning-based classification. The primary features identified as significant indicators of stress patterns include humidity, temperature, and step count. Each record is labeled into one of three distinct stress categories: No Stress (0), Mild Stress (1), and High Stress (2). It is important to note that the stress labels in this dataset are not independently clinically validated and are likely derived from the same input features (humidity, temperature, and step count). As a result, the dataset reflects a feature-dependent labeling mechanism rather than an externally established ground truth (e.g., physiological or psychological assessment).

The specific feature descriptions are as follows:

- **Humidity:** Represents the sweat level of an individual; as physiological stress increases, body temperature rises, triggering sweat gland activity.
- **Temperature:** Refers to the body temperature of the individual recorded during stressful or non-stressful states.
- **Step count:** Measures the physical activity level, represented by the number of steps covered by the individual during the observation period.
- **Stress level:** The target variable inferred based on the interaction of the three aforementioned factors, rather than independently measured clinical stress.

A preliminary analysis of the dataset was conducted to evaluate class distribution and ensure balanced learning. The dataset contains 680 samples of No Stress (34.0%), 712 samples of Mild Stress (35.6%), and 609 samples of High Stress (30.4%). This relatively even distribution prevents the model from developing a bias toward any specific class, ensuring the Attention-Enhanced Hypergraph Neural Network (AE-HGNN) can learn representative patterns across all stress levels. Given the feature-derived nature of the labels, the dataset is used in this study primarily as a benchmark to evaluate the capability of the proposed AE-HGNN model in learning complex feature interactions, rather than as a definitive representation of real-world stress prediction. The preprocessing pipeline is critical for preparing the structured data for hypergraph construction (Pan et al., 2024). Initially, the raw data is loaded and formatted to align environmental attributes with their corresponding labels. To enhance data quality, outlier detection is performed using both Z-Score analysis and the Interquartile Range (IQR) method. Specifically, we applied a Z-score threshold of |*z*|>3 and the 1.5 × IQR rule across all numerical features to identify anomalous signals. This dual-verification confirmed that no samples reached the outlier threshold (union = 0), ensuring the raw dataset accurately captures the experimental range without skewing the threshold-based hyperedge formation. Following this verification, the features are normalized to a common scale, providing a robust input for the subsequent hypergraph modeling phase. Table 2 presents a representative sample of the dataset using raw (physical) values along with min-max scaled values.

**Table 2:** Dataset sample (raw and min-max scaled values).

Sr. No.	Humidity (raw)	Temperature (raw)	Step count	Humidity (minmax)	Temperature (minmax)
1	26.85	95.85	169	0.8425	0.8425
2	28.43	97.43	185	0.9215	0.9215
3	25.57	94.57	183	0.7785	0.7785
4	28.40	97.40	182	0.9200	0.9200

Algorithm 1 Data preprocessing. 
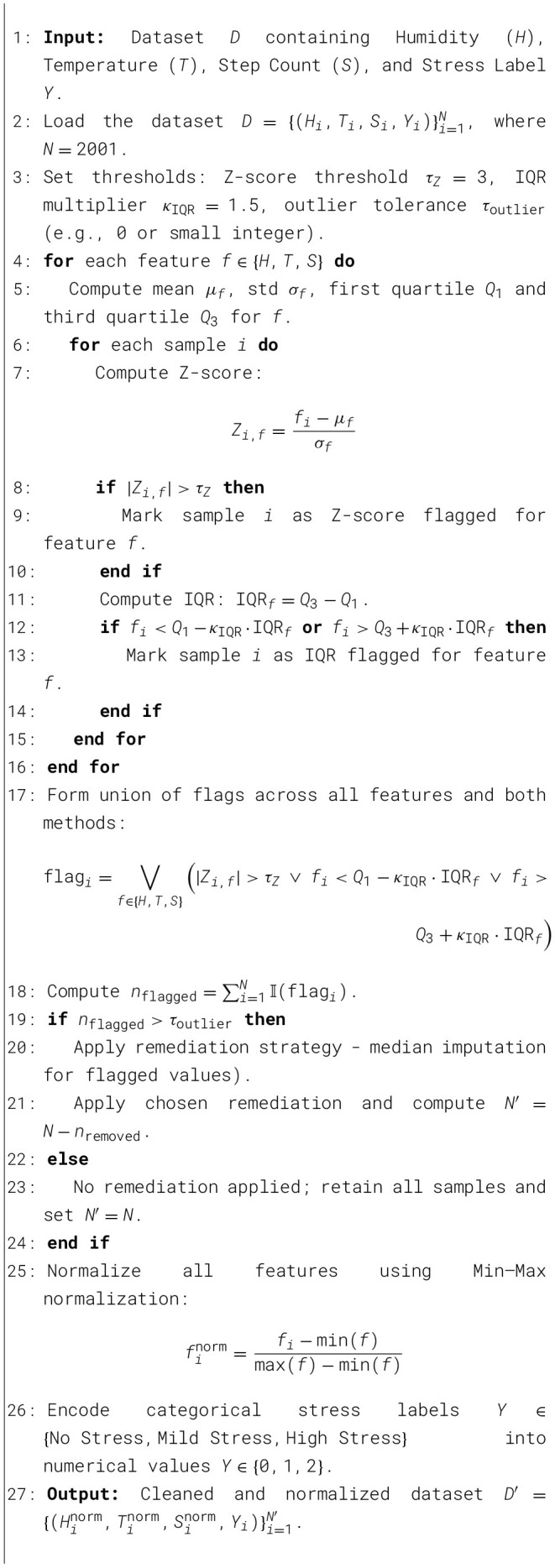

For the Stress-Lysis dataset used in this study, the implemented checks (Z-score |*z*|>3 and IQR 1.5 × IQR) flagged *n*_flagged_ = 0 rows (union = 0). Therefore, no records were removed and *N*′ = *N* = 2, 001.

### 3.2 Feature distributions and correlations

**Figure 2:**
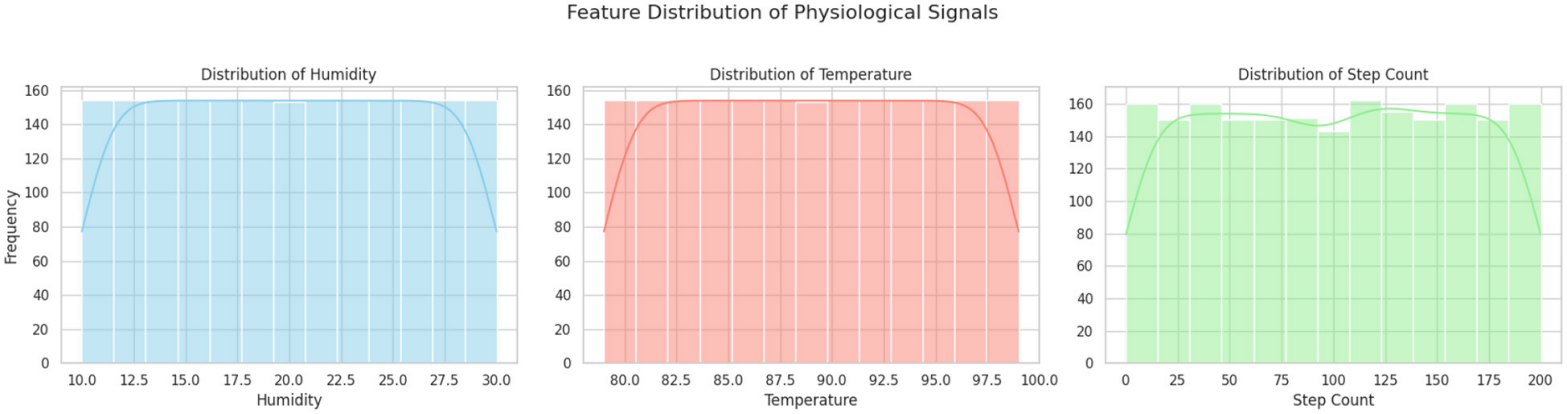
Feature distribution of Humidity, Temperature, Step count.

The distribution of the Humidity, Temperature and Step Count feature is illustrated in Figure 2. The histogram reveals a multimodal distribution with several distinct peaks, indicating that the humidity values are clustered around specific levels rather than being spread uniformly. The distribution of Temperature, mirrors this pattern, exhibiting a similar multimodal shape that suggests a strong relationship with humidity. In contrast, the Step count data displays a nearly uniform distribution. This indicates that the dataset contains a wide and balanced range of movement data, ensuring the model is trained on diverse activity levels.

**Figure 3:**
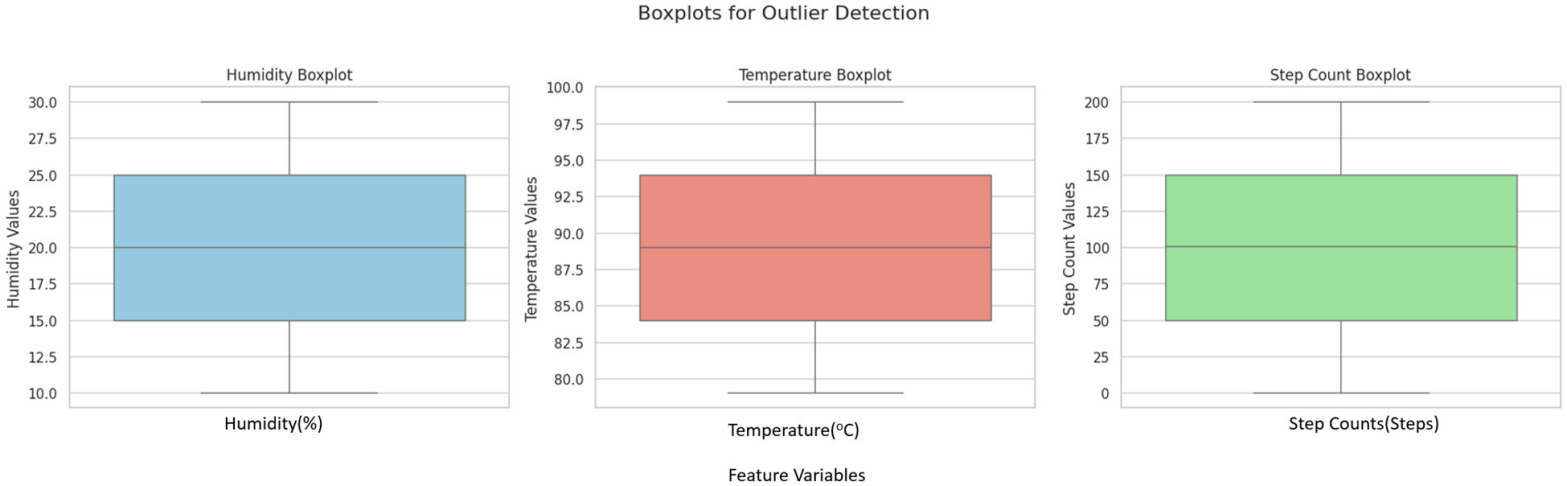
Boxplots for Humidity, Temperature, and Step Count.

A boxplot analysis was conducted to detect outliers, as shown in Figure 3. The plots for Humidity, Temperature, and Step count all show symmetrical distributions with no data points extending beyond the whiskers. To validate this visual inspection, we performed formal outlier checks using a Z-score threshold of |*z*|>3 and the IQR rule with multiplier 1.5 × IQR across all numeric features; the union of these checks flagged 0 samples (union = 0). This clean result confirms the absence of extreme values or outliers, demonstrating the high quality of the data and obviating the need for outlier removal before model training.

**Figure 4:**
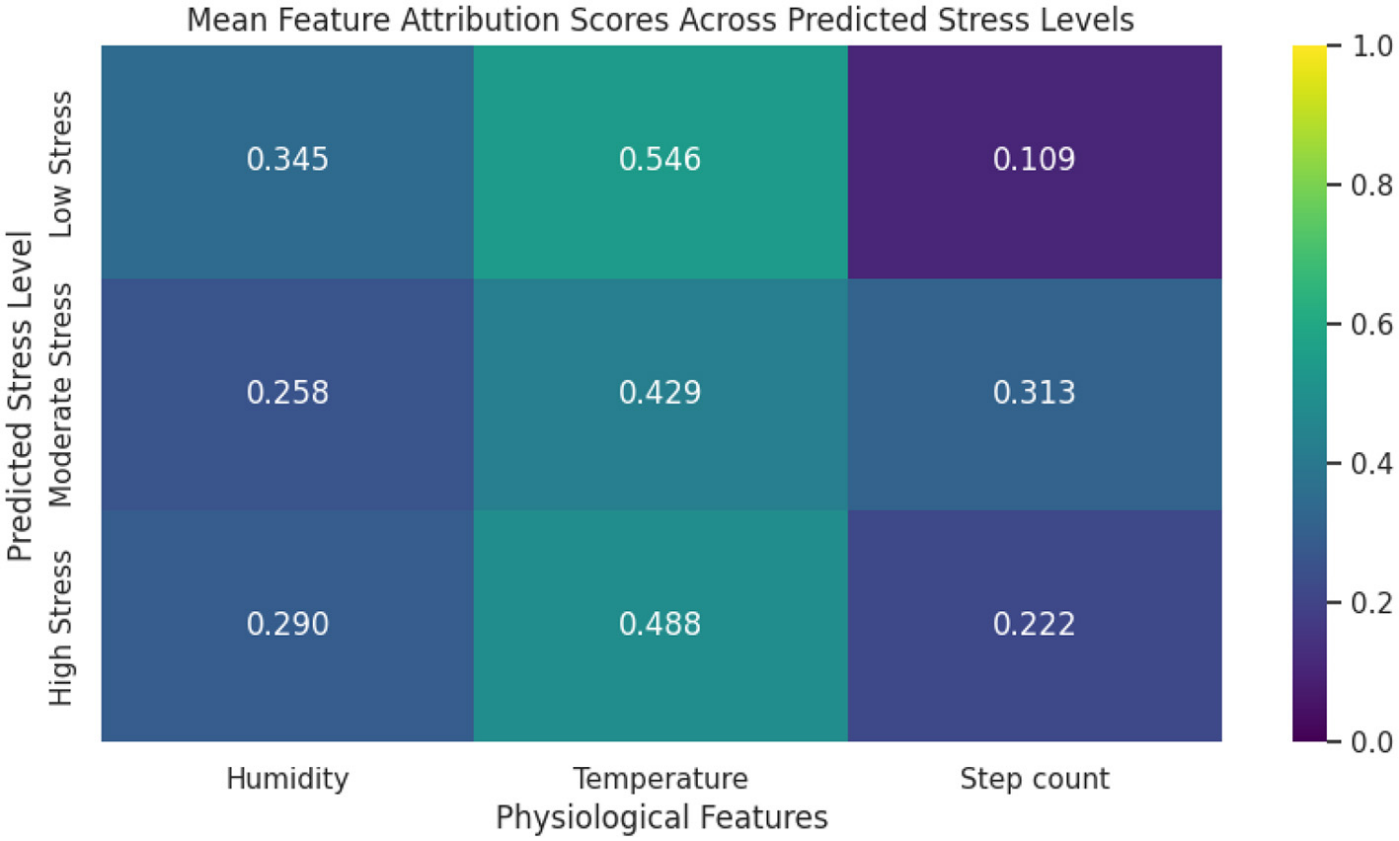
Mean Feature Attribution scores across predicted stress levels based on the AE-HGNN attention mechanism.

Figure 4 illustrates the mean feature attribution scores derived from the learned attention weights of the AE-HGNN model across the three predicted stress categories. These scores reflect the internal weighting assigned by the attention mechanism to the physiological features (Humidity, Temperature, and Step count) during inference. As shown in the heatmap, Temperature consistently receives the highest mean attribution scores (ranging from 0.429 to 0.546) across all stress levels, suggesting it is a primary driver for the model's decision-making process. Humidity follows as the second most influential feature, particularly for Low Stress predictions (0.345), while Step count contributes moderately across categories. While these attention weights provide a mechanism for model interpretability, we acknowledge that attention attribution does not directly imply clinical or causal feature importance. Instead, they represent the model's focus on specific input signals to optimize predictive accuracy. To validate these internal insights, we performed a *post-hoc* sensitivity analysis using permutation importance on the hold-out test set, which corroborated the ranking of Temperature as the most significant predictor for the Stress-Lysis dataset classification tasks.

Algorithm 2 Hypergraph generation using threshold-based similarity. 
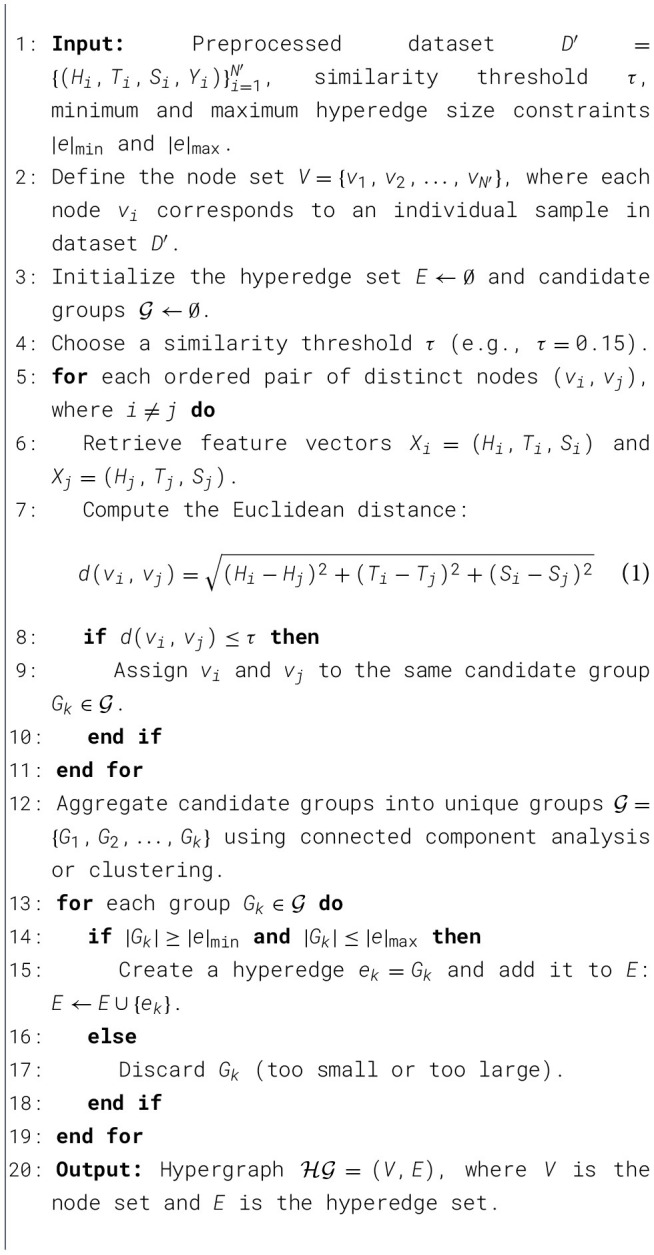

### 3.3 Hypergraph construction

After data preprocessing, a hypergraph structure is constructed in which each individual sample is represented as a node, and higher-order relationships are modeled through hyperedges. Unlike conventional Graph Neural Networks (GNNs) that capture only pairwise interactions between nodes, the Hypergraph Neural Network (HGNN) framework connects multiple nodes simultaneously, enabling the modeling of complex group-level dependencies among individuals. In the proposed approach, nodes are grouped into hyperedges based on the similarity of their environmental and behavioral conditions, specifically humidity, temperature, and step count. To identify individuals with comparable patterns, a threshold-based similarity strategy is employed. For each pair of nodes, the Euclidean distance between their feature vectors is computed, and nodes with distances below a predefined threshold are assigned to the same candidate group. These candidate groups are then aggregated to form hyperedges, effectively capturing higher-order interactions among multiple samples. The detailed procedure for threshold-based hypergraph generation, including node grouping, hyperedge formation, and pruning of excessively small or large hyperedges, is formally described in Algorithm 2. This hypergraph construction strategy provides a structured foundation for the subsequent attention-enhanced hypergraph convolution, allowing the model to exploit multi-node dependencies for improved stress classification.

### 3.4 Attention enhanced HGNN model architecture

**Figure 5:**
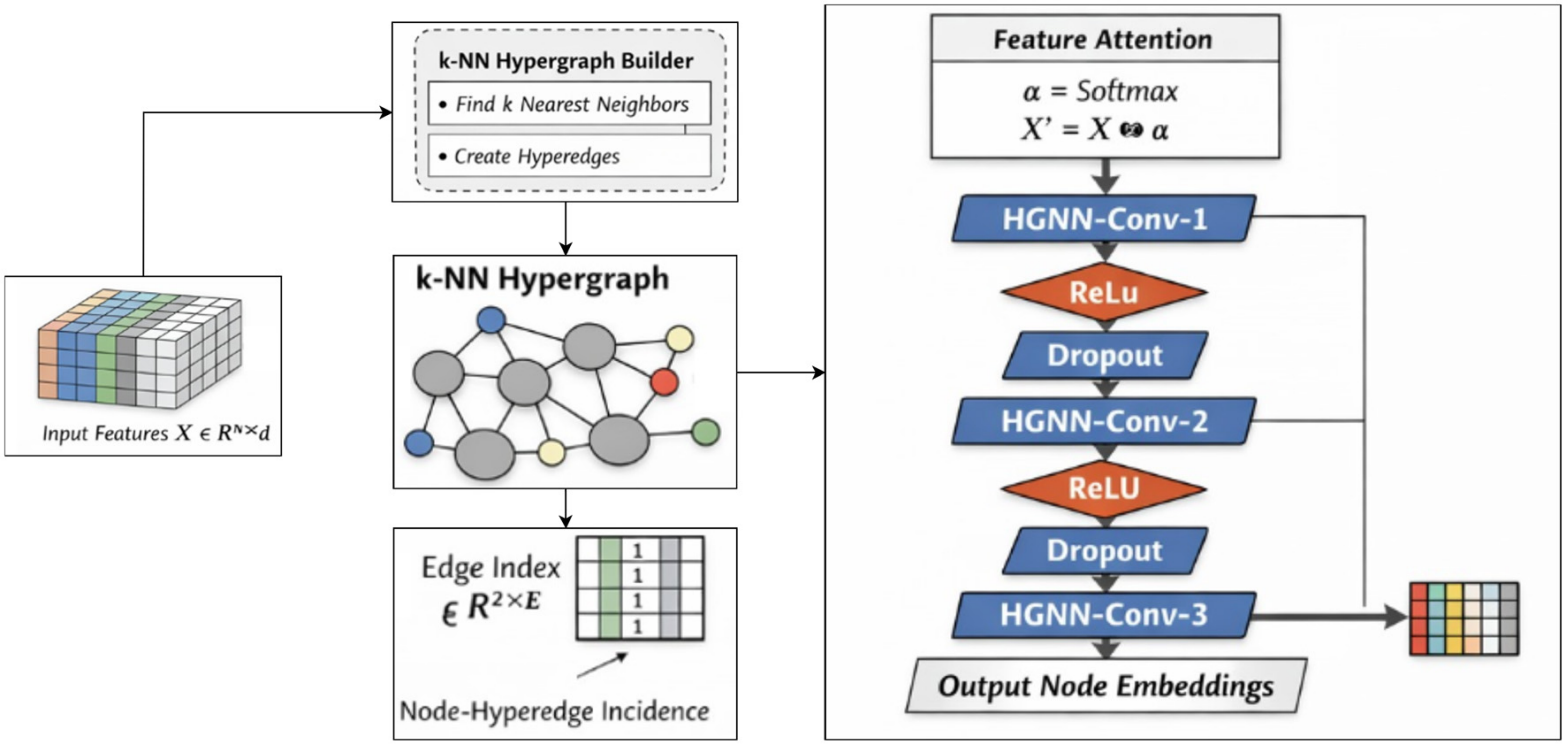
Architecture of proposed model.

The main structure of the system is the AE-HGNN (Attention Enhanced Hypergraph Neural Network), model architecture shown in Figure 5, that aims to learn high-order relationships but selectively concentrate on the most informative nodes and hyperedges. The input feeds the data which is then presented as a feature matrix in the input layer. The Attention-augmented Hypergraph Convoluted Layers (AHGCN) are applied to the hypergraph structure by not only aggregating information relayed by the nodes tied by hyperedges, but also allocating attention weights to highlight more significant node-hyperedge interactions.

The AE-HGNN captures higher-order node interactions to learn representations (in contrast to typical GNNs that only capture pairwise relationships). This is further improved by a mechanism of attention where connections are dynamically weighted which enables the model to optimize on complex dependencies and interpretability. After these refined representations, they are sent through fully connected layers where the activation function of ReLU adds non-linearity. Lastly, a SoftMax layer conducts the classification task, by producing probabilities in stress-level category. The combination of attention mechanisms with the hypergraph convolutions not only enhances the performance and accuracy of classifications, but also allows explanation by means of attention heatmaps, which demonstrate which features and hyperedges have the most significant contribution to the predictions.

#### 3.4.1 Working of attention enhanced hypergraph convolutional layer

The input to the Attention-Enhanced Hypergraph Convolutional Layer (AE-HGCN) is a feature matrix, with environmental and behavioral data (humidity, temperature, and step count), and an adjacency matrix that describes hyperedges linking individuals. AE-HGNN builds on the standard hypergraph convolution by incorporating an attention mechanism, which dynamically adjusts the importance of different nodes and hyperedges during the feature aggregation process. This allows the model to emphasize more informative interactions while suppressing less relevant ones.

Inside the attention enhanced hypergraph convolution the transformation process is as follows:

1. Feature aggregation with attention: AE-HGCN does not equally balance the weights among all nodes in a hyperedge, but instead it learns attention coefficients, which imply the relative importance of each node or hyperedge. This allows the model to learning small yet significant dependencies (e.g., the high-activity temperature spikes are more important when it comes to detecting stress).
2. Nonlinear activation: After the attention-weighted convolution operation is applied, the ReLU activation function is used to introduce non-linearity, ensuring that only significant features are retained.
3. Layer stacking: Multiple AE-HGCN layers are stacked, where each layer refines the attention distribution and progressively learns higher-order contextual representations from the data. This deepens the model's ability to capture complex stress-related patterns.
4. The mathematical representation of the attention enhanced hypergraph convolution operation is given in Equation 2 (Feng et al., 2019; Yadati et al., 2019):

$$\begin{array}{l} {\text{H}}^{\left(l+1\right)}=\sigma \left({\text{D}}_{v}^{-\frac{1}{2}}\alpha ⊙\left({\text{H}}^{\left(l\right)}{\text{D}}_{e}^{-1}{\text{H}}^{\left(l\right)⊤}\right){\text{D}}_{v}^{-\frac{1}{2}}\text{W}\right) \end{array}$$ (2)

**where**

- **H**^(*l*)^ is the node feature matrix at layer *l*.
- **D**_*v*_ and **D**_*e*_ are the degree matrices of nodes and hyperedges, respectively.
- **W** represents trainable weight parameters.
- σ(·) is the activation function (ReLU) applied after feature transformation.
- ⊙ denotes element-wise attention weighting operation.
- **α** denotes the learned attention coefficients over nodes and hyperedges.

Through incorporating attention in the convolution, AE-HGNN is more discriminative in its learning. The last refined feature matrix emphasizes patterns of stress and underemphasizes noisy or less useful signals resulting in a better classification accuracy and interpretability than the vanilla HGNN.

#### 3.4.2 Fully connected layers and activation functions

Once all Attention-Enhanced Hypergraph Convolutional Layers (AE-HGCN) pass, the attention-weighted features extracted from the hypergraph are flattened and introduced to fully connected layers. These layers transform the high-dimensional attention-informed embeddings into compact decision boundaries for classification. The final stress level prediction is obtained using the SoftMax activation function, which computes the probability distribution across the stress categories—No Stress, Mild Stress, and High Stress - are calculated using the SoftMax activation function and Equation 3 (Bishop, 2006):


$$\begin{array}{l} P\left(y=k|\text{x}\right)=\frac{e^{z_{k}}}{\underset{j}{\sum }e^{z_{j}}} \end{array}$$ (3)

**where** *z*_*k*_ is the input to the softmax function for class *k*. The denominator ensures that the outputs sum to one, allowing them to be interpreted as probabilities.

#### 3.4.3 Loss function and optimization

The degree of discrepancy between the model's anticipated and actual values for the stress labels is indicated by the cross entropy loss function, which also helps the model produce more accurate predictions. Equation 4 is used to calculate the loss (Bishop, 2006):


$$\begin{array}{l} L=-\overset{N}{\underset{i=1}{\sum }}\overset{K}{\underset{j=1}{\sum }}y_{ij}\log\left({ŷ}_{ij}\right) \end{array}$$ (4)

**where**

- *N* is the total number of samples.
- *K* represents the number of stress categories.
- *y*_*ij*_ is the ground-truth label for sample *i* belonging to class *j*.
- ŷ_*ij*_ is the predicted probability for class *j*.

Algorithm 3 Proposed attention-enhanced hypergraph neural network (AE-HGNN). 
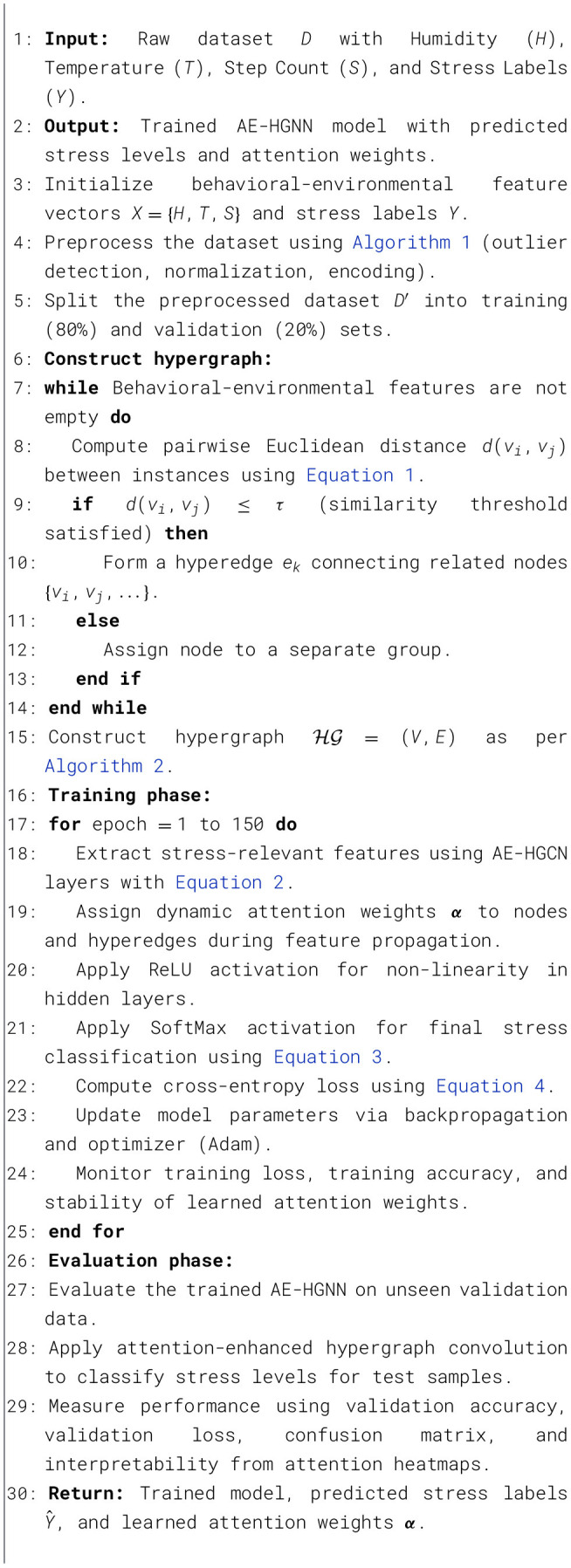

### 3.5 Model training, evaluation, and prediction

The Attention-Enhanced Hypergraph Neural Network (AE-HGNN) is trained and evaluated by leveraging multinode dependencies and attention-based weighting of hyperedges to ensure robust generalization across diverse stress patterns. To mitigate overfitting and validate model stability, a 5-fold cross-validation strategy is employed, where the preprocessed behavioral environmental data is partitioned into training and validation sets using an 80:20 split. During the training phase, higher order stress features are extracted through AE-HGCN layers, where the integrated attention mechanism dynamically prioritizes the most informative nodes and hyperedges, followed by ReLU activation for non-linearity and a SoftMax layer to generate class probabilities. Convergence is monitored by documenting training accuracy, loss, and attention weight distributions after each epoch. In the evaluation and testing phase, the trained model is applied to unseen validation data using the attention-enhanced convolutional process to predict stress levels. Model reliability is assessed through validation loss and accuracy, while the final output layer classifies individuals into No Stress, Mild Stress, or High Stress categories. To justify these predictions and ensure clinical interpretability, the system generates a comprehensive classification report including precision, recall, F1-score, and overall accuracy alongside confusion matrices and attention heatmaps that highlight the specific signals, such as humidity spikes or step count variations, that most significantly influenced the classification.

## 4 Results and discussion

The model obtained validation results through cross-validation that showed superior accuracy levels compared to baseline methods while demonstrating statistical significance at (*p* < 0.01). The learning rate of 0.01 was selected as it provided stable convergence without oscillations, and a dropout rate of 0.5 was used for reducing overfitting risk while retaining stress-relevant representations. The primary reason for choosing the Attention Enhanced-HGNN over traditional GNNs and standard HGNNs is its feature attention mechanism, which learns the relative importance of each input signal (humidity, temperature, and step count) and thereby improves classification performance, achieving a test accuracy of 99.75%. The best-performing configuration is obtained with *k* = 5, hidden dimension = 128, and learning rate = 0.01, and the corresponding learned attention weights for the best model indicate higher contribution from humidity (α = 1.3310), followed by temperature (α = 0.9829) and step count (α = 0.7144), highlighting the model's ability to provide interpretable feature importance for stress prediction.

**Figure 6:**
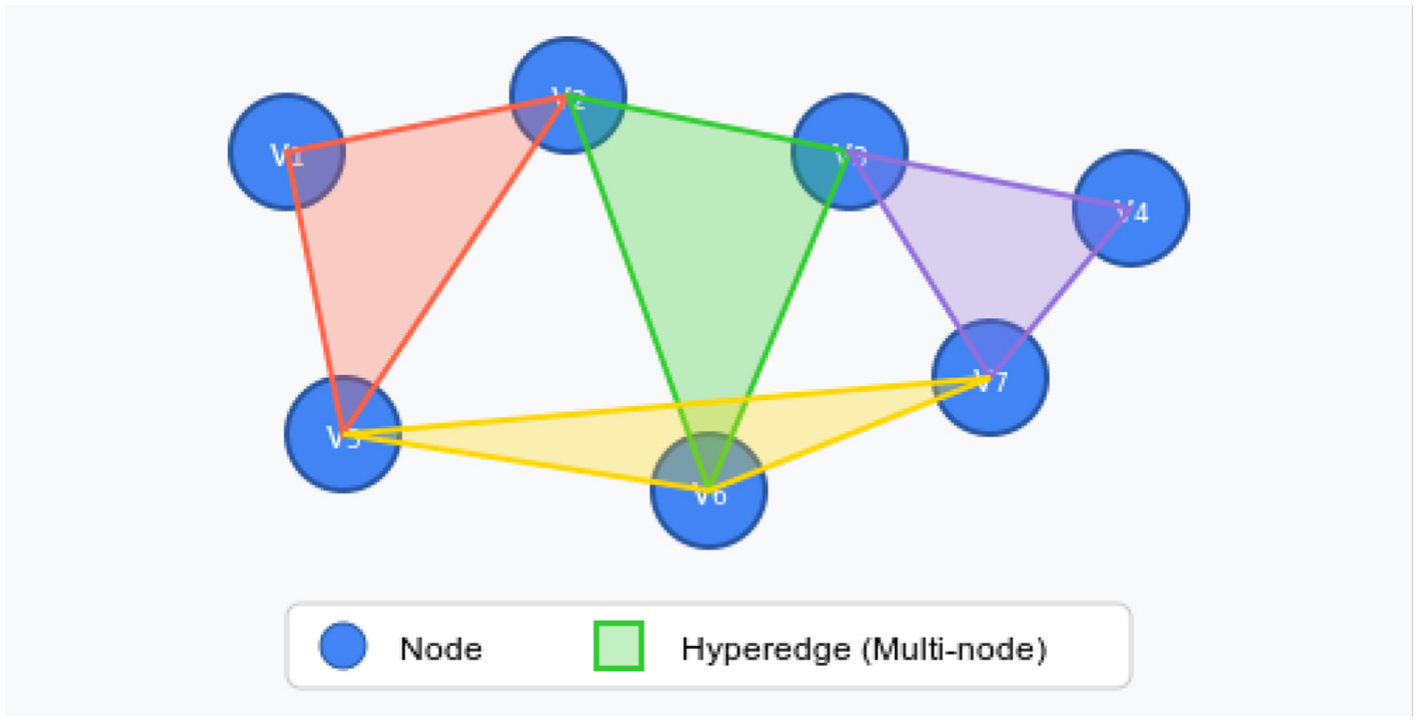
Hypergraph multi node relationship.

**Figure 7:**
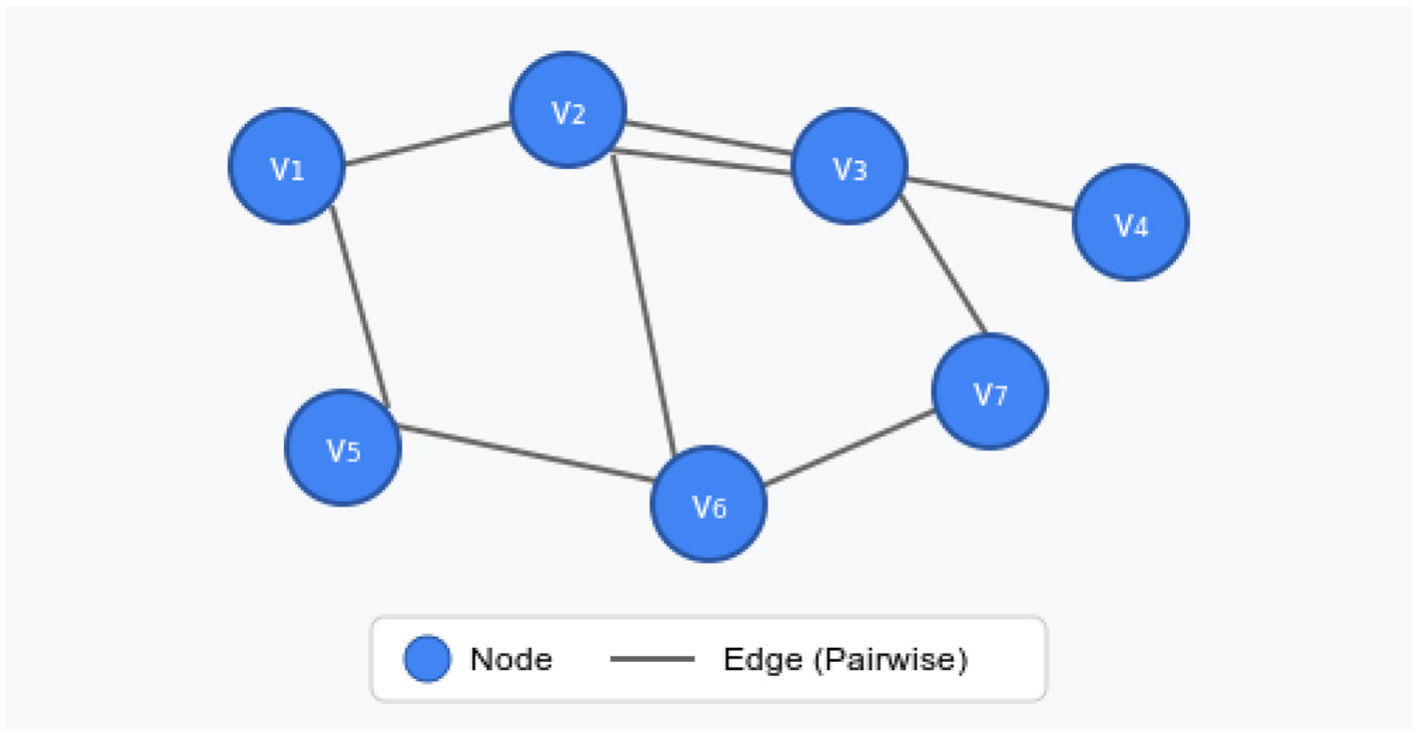
GNN pairwise connection.

A hyperedge is formed when the distance between two persons is less than a predefined threshold. This allows the model to learn complex, multi node dependency which is more effective in stress classification. Figures 6, 7 shows how individuals are interconnected within hyperedges, Highlighting the difference between conventional GNN pairwise connection and hypergraph based multi node relationship.

### 4.1 Experimental setup

The experimental evaluation of the proposed Attention-Enhanced Hypergraph Neural Network (AE-HGNN) for stress prediction was conducted on a dedicated computational environment to ensure consistency and reproducibility. The system was implemented using the Python programming language and leveraged state-of-the-art deep learning (Goud and Garg, 2023) and graph processing libraries. The experiments were structured into two primary stages: a training phase to optimize the model parameters and a testing phase to evaluate generalization performance on unseen data. The detailed hardware specifications, software environment, and model training configurations are summarized in Table 3. Complete Model is shown in Algorithm 3.

**Table 3:** Experimental setup and model configuration.

Category	Specifications/parameters
Hardware	Processor: Ryzen 5 5600H GPU: AMD Radeon 5500M RAM: 8GB DDR4, Storage: 512GB SSD
Software stack	Language: Python 3.10 Framework: PyTorch 2.1.0, PyG 2.3.0 Libraries: DeepHypergraph 1.2.0, Pandas, NumPy
Data configuration	Training/Testing Split: 80:20 Cross-Validation: 5-Fold
Hyperparameters	Learning Rate: 0.01, Epochs: 150 Dropout Rate: 0.5, Hidden Dimension: 128
Architecture	Activation: ReLU (Hidden), SoftMax (Output) Loss function: Cross-entropy
Metrics	Accuracy, Precision, Recall, F1-score

### 4.2 Results and analysis of proposed model

**Figure 8:**
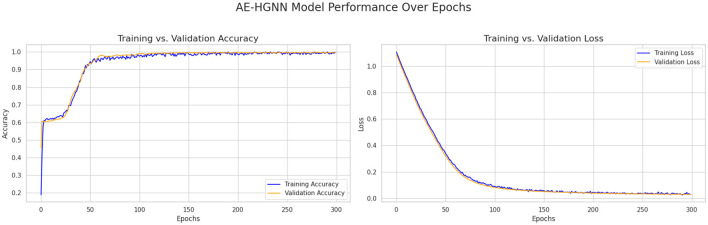
Training vs. validation accuracy and loss.

Figure 8 shows the AE-HGNN model's training and validation performance on the Stress Lysis dataset. The training and validation accuracy curves both converge very closely, reaching around 99.6% training accuracy and 99.75% testing accuracy after 150 epochs. Accuracy rises steeply during the first 20 epochs and then stabilizes, indicating that the model learns quickly and maintains strong generalization on unseen data. The loss curves also follow a smooth decreasing trend. Both the training and validation losses start at approximately 1.1–1.2 and consistently decline, reaching final values of around 0.05 for training loss and 0.06 for validation loss. The near overlap of training and validation loss demonstrates that the AE-HGNN avoids overfitting and successfully minimizes errors during learning. This behavior validates the effectiveness of the attention-enhanced hypergraph convolutional layers in capturing higher-order relationships while preserving generalization capability.

**Figure 9:**
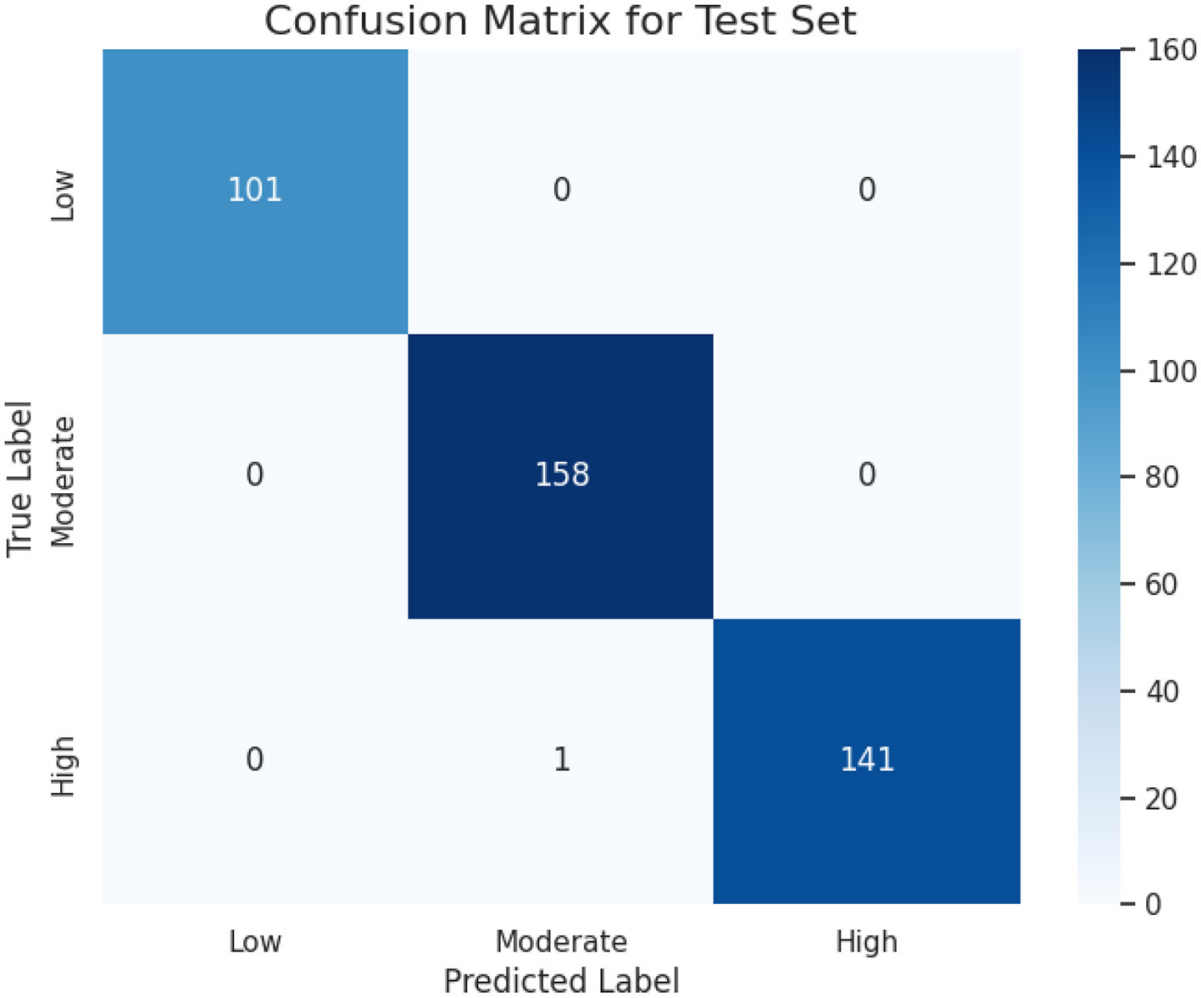
Confusion matrix for proposed HGNN model.

The confusion matrix of the Stress Lysis dataset after classification using the AE-HGNN model, as illustrated in Figure 9, summarizes the correctly and incorrectly identified instances across the three stress categories: Low, Moderate, and High. Correct classifications are represented along the diagonal, while misclassifications are indicated by off-diagonal values. Specifically, the model correctly classifies 101 Low stress samples, 158 Moderate stress samples, and 141 High stress samples, with minimal misclassifications observed only where 1–2 High stress samples were predicted as Moderate. Notably, no Low stress or Moderate stress samples were misclassified, and this near-perfect diagonal structure demonstrates that the Attention-Enhanced HGNN effectively separates stress levels with extremely low error rates, further confirming the robustness and high discriminative power of the proposed model.

**Figure 10:**
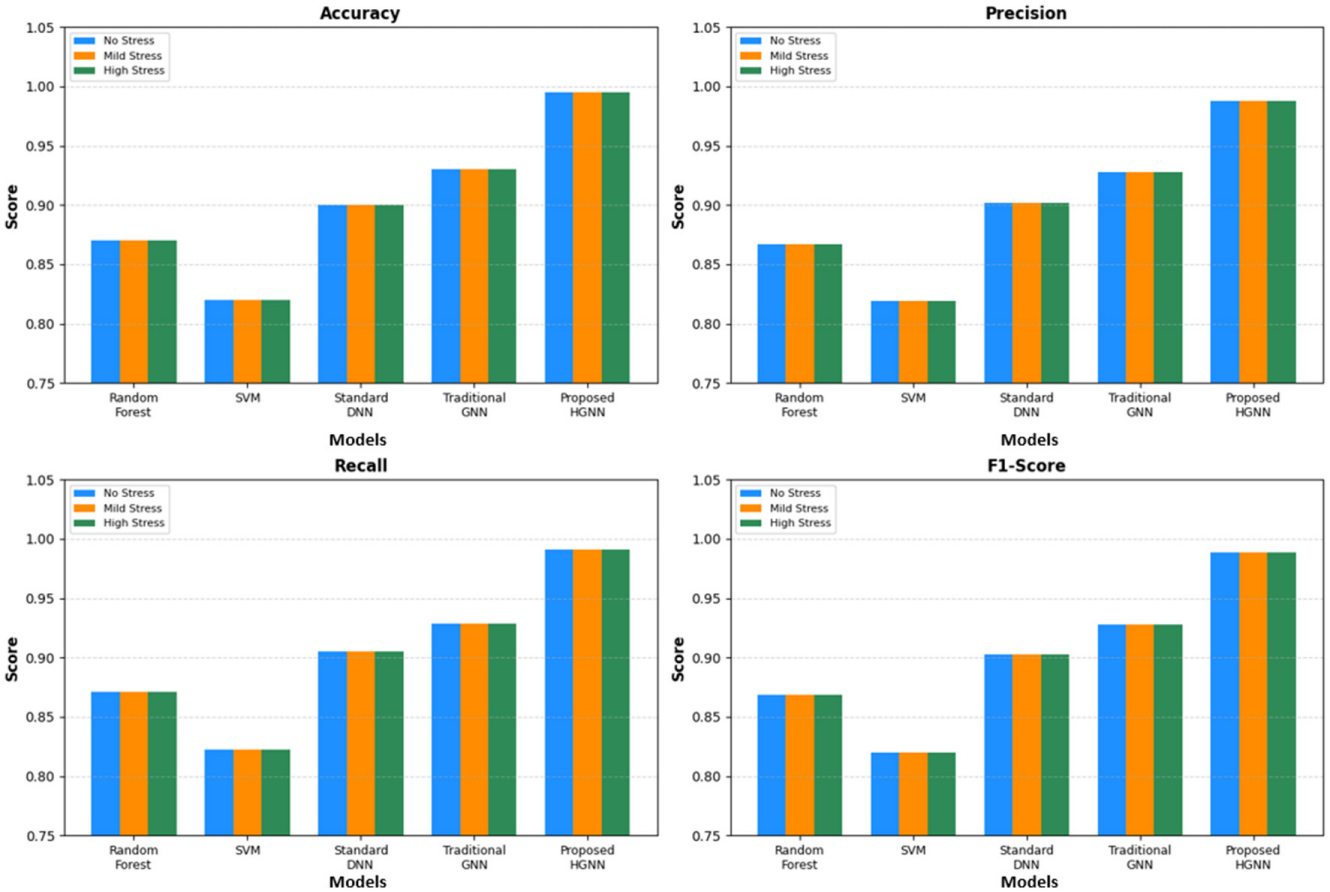
Comparison of model performance across stress categories.

The proposed AE-HGNN model is evaluated using standard classification metrics, including accuracy, precision, recall, and F1-score, which jointly quantify overall correctness, reliability of positive predictions, coverage of true positives, and the balance between precision and recall, respectively (Kasar et al., 2025; Gaurav et al., 2023). These metrics are computed from the confusion matrix terms: true positives (TP), true negatives (TN), false positives (FP), and false negatives (FN). Figure 10 illustrate the comparative analysis of accuracy, precision, recall, and F1-score across baseline models and the proposed AE-HGNN, while the consolidated numerical results, including training time, are reported in Table 4. As shown in Table 4, AE-HGNN achieves the best overall performance across all metrics, demonstrating both strong discriminative capability and consistent classification behavior across stress categories.

**Table 4:** Performance metrics of different models.

Model	Accuracy (%)	Precision (%)	Recall (%)	F1-score (%)	Training time (s)
Random forest	87.3 ± 1.2	86.7 ± 1.4	87.1 ± 1.3	86.9 ± 1.2	12.3
SVM	82.8 ± 1.5	81.9 ± 1.7	82.2 ± 1.6	82.0 ± 1.5	15.7
Standard DNN	90.7 ± 1.1	90.2 ± 1.3	90.5 ± 1.2	90.3 ± 1.2	45.2
Traditional GNN	93.1 ± 0.8	92.8 ± 0.9	92.9 ± 0.8	92.8 ± 0.9	67.8
Proposed AE-HGNN	99.75 ± 0.2	98.8 ± 0.5	99.1 ± 0.4	98.9 ± 0.4	73.4

**Figure 11:**
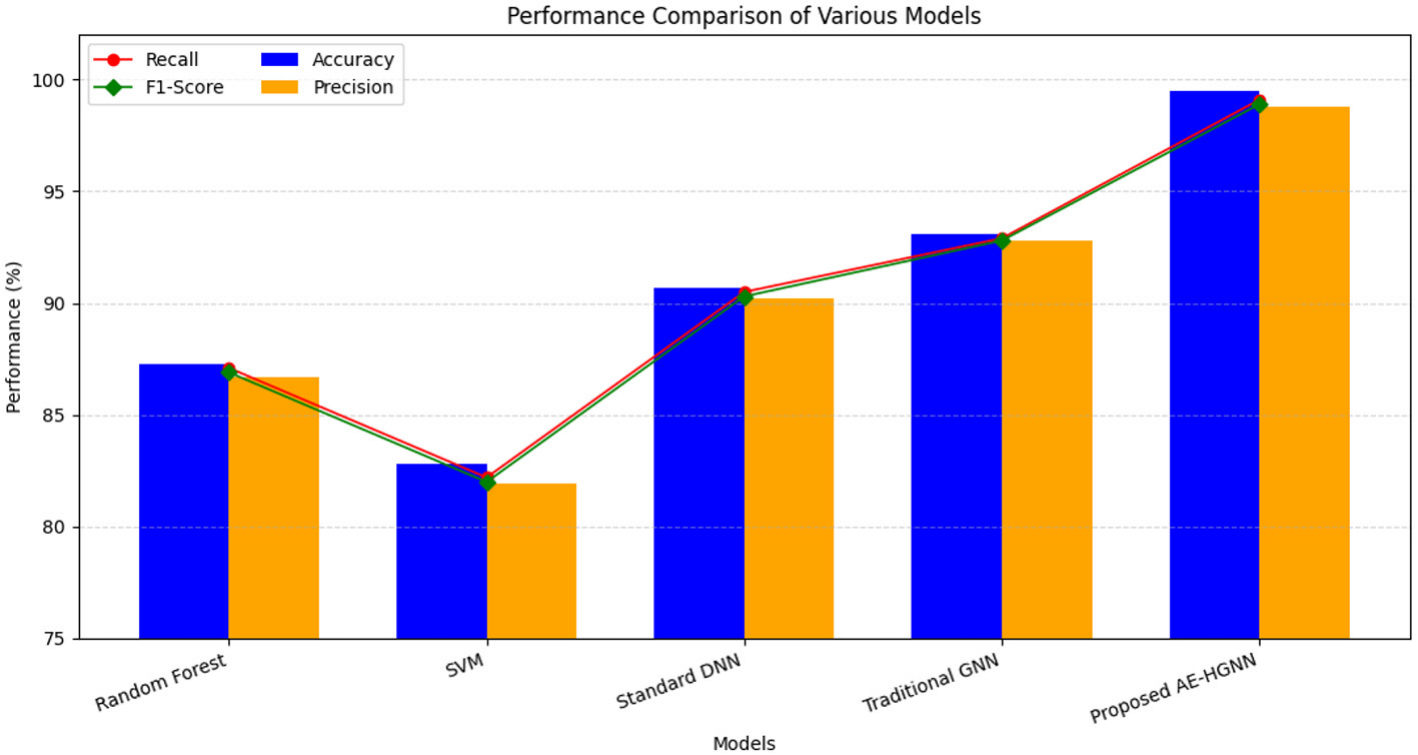
Performance evaluation comparison.

Figure 11 presents a mixed-chart comparison of model performance, where the proposed AE-HGNN achieves state-of-the-art results across all metrics, significantly surpassing baseline methods including Random Forest (87.3%), SVM (82.8%), Standard DNN (90.7%), and Traditional GNN (93.1%).

### 4.3 Robustness and generalization analysis

To evaluate the robustness and generalization capability of the proposed AE-HGNN model, a rigorous experimental protocol was adopted using stratified data partitioning, five-fold cross-validation, and an independent hold-out test set. The dataset was first divided into a train+validation subset containing 1,600 samples and a hold-out test subset containing 401 samples. The class distribution in the hold-out test set was preserved as 101 samples for *No Stress*, 158 samples for *Mild Stress*, and 142 samples for *High Stress*. All feature normalization steps were performed after data partitioning to ensure that validation and test samples remained unseen during preprocessing.

The cross-validation performance of AE-HGNN is summarized in Table 5. Across the five folds, the model achieved a mean accuracy of 98.44 ± 0.56%, mean precision of 98.30 ± 0.77%, mean recall of 98.37 ± 0.40%, and mean F1-score of 98.32 ± 0.60%. The area under the ROC curve (one-vs.-rest, macro) across folds was 99.96% (mean ROC AUC = 0.99, fold-wise SD = 0.00031, bootstrap 95% CI = [0.99, 1.00]), and Cohen's Kappa averaged 0.9741 (fold-wise SD = 0.0138, 95% CI = [0.95, 0.99]). The corresponding 95% confidence intervals reported in Table 5 further indicate that the performance remains highly stable across different data splits. The narrow intervals confirm that the proposed model is not sensitive to a specific partition of the dataset and maintains consistent predictive behavior.

**Table 5:** Five-fold cross-validation performance of AE-HGNN.

Metric	Mean	SD	95% CI
Accuracy	98.44%	0.56%	[97.66%, 99.21%]
Precision	98.30%	0.77%	[97.24%, 99.36%]
Recall	98.37%	0.40%	[97.81%, 98.93%]
F1-score	98.32%	0.60%	[97.49%, 99.15%]
ROC AUC	99.96%	0.03%	[99.93%, 100.0%]
Cohen's Kappa	0.9741	0.0138	[0.9570, 0.9913]

**Figure 12:**
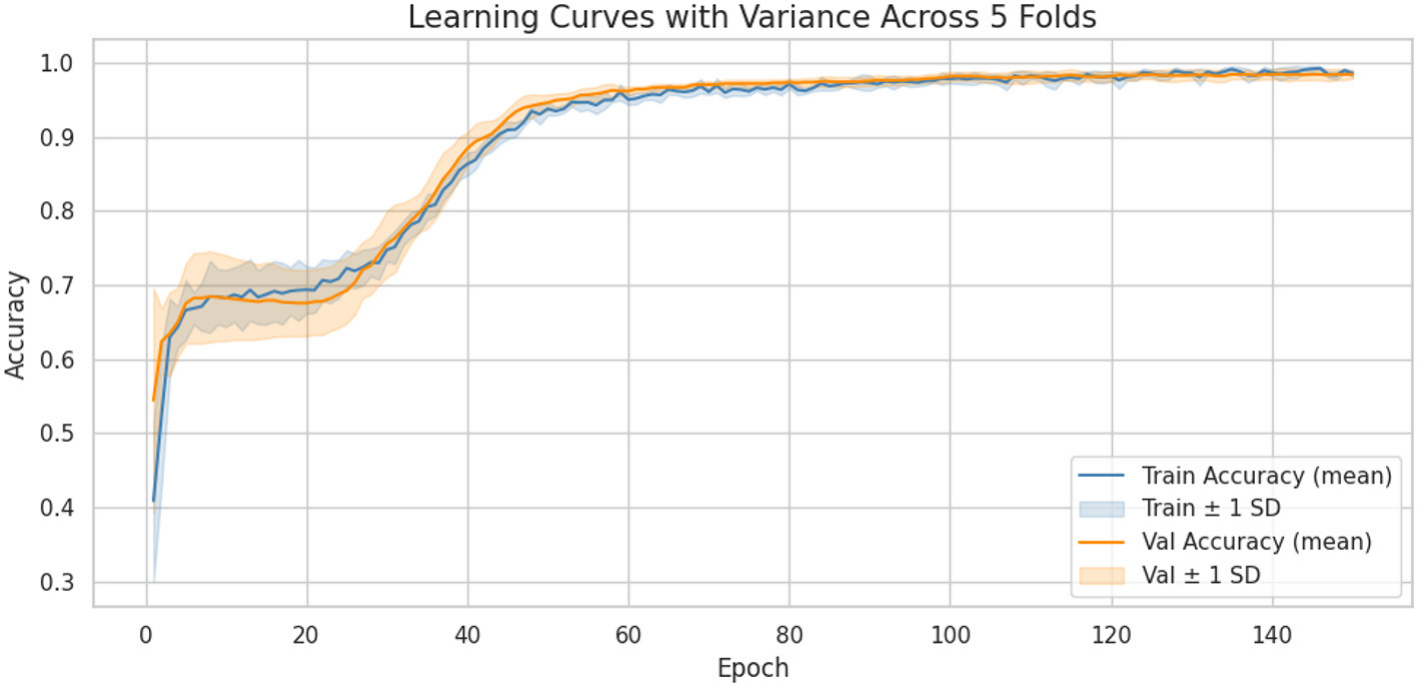
Training and validation learning curves of AE-HGNN across five cross-validation folds.

**Figure 13:**
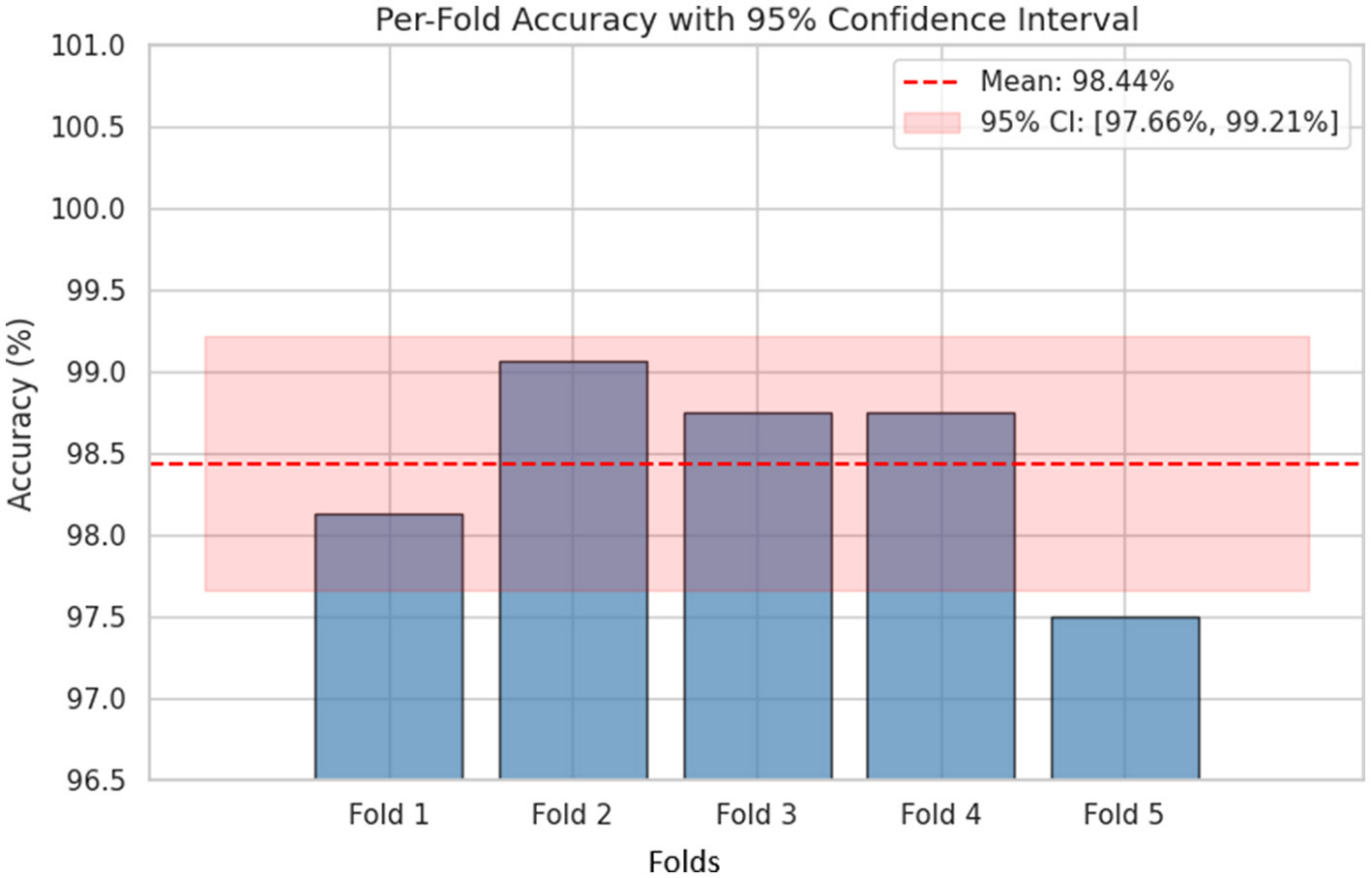
Per-fold accuracy with confidence interval.

The learning dynamics of the model are illustrated in Figure 12. As shown in Figure 12, the training and validation trajectories converge smoothly with limited variance across folds, indicating stable optimization behavior throughout the training process. The close agreement between the fold-wise curves further suggests that the network learns discriminative physiological representations in a reliable and repeatable manner. In addition, the fold-wise validation accuracy with the corresponding confidence interval is presented in Figure 13. The small variation in accuracy across folds shown in Figure 13 further confirms the consistency and robustness of the proposed AE-HGNN model.

**Figure 14:**
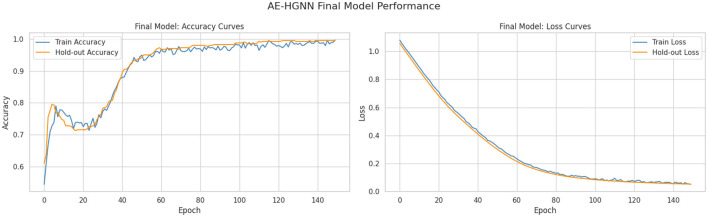
Training and hold-out accuracy and loss curves across epochs.

**Figure 15:**
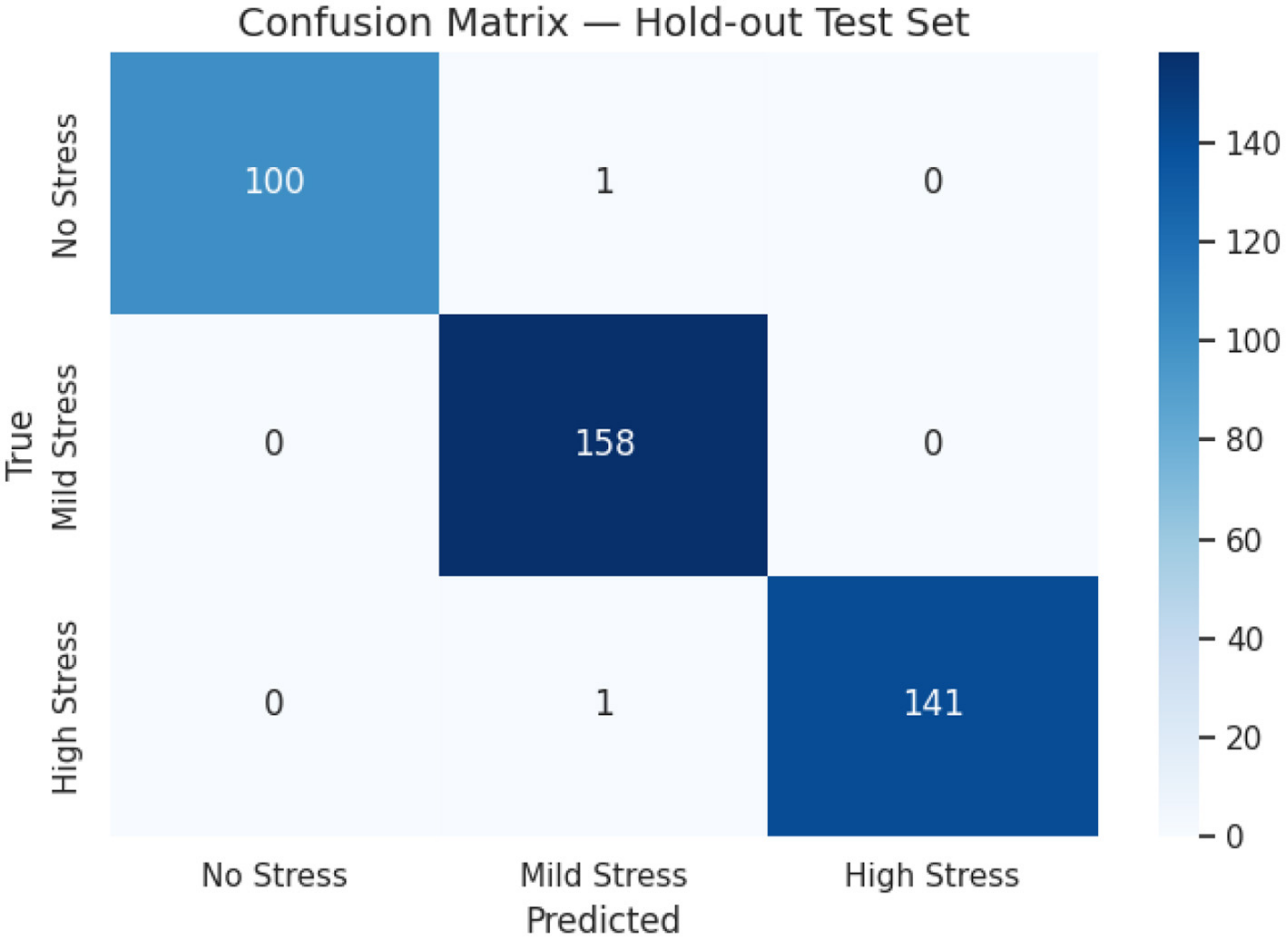
Confusion matrix of AE-HGNN on the independent hold-out test set.

To further examine the practical generalization ability of the model, the final AE-HGNN was trained on the complete train+validation subset and evaluated on the independent hold-out test set. The overall hold-out results are reported in Tables 6 and 7. The model achieved a hold-out accuracy of 99.50% and a macro F1-score of 99.51%, which are closely aligned with the cross-validation performance reported in Table 5. This agreement between cross-validation and hold-out evaluation demonstrates that the proposed framework generalizes well to unseen samples. The epoch-wise training and hold-out performance trends are shown in Figure 14. As can be observed from Figure 14, both the accuracy and loss curves exhibit stable convergence behavior, with training and hold-out trends remaining closely aligned throughout the optimization process. This behavior indicates effective learning without noticeable instability during training. A more detailed class-wise analysis on the hold-out set is presented in Table 8. The model attained precision, recall, and F1-scores close to 1.00 for all three stress categories. In particular, the *No Stress* class achieved a precision of 1.00, recall of 0.99, and F1-score of 1.00, while the *Mild Stress* class achieved a precision of 0.99, recall of 1.00, and F1-score of 0.99. Similarly, the *High Stress* class obtained a precision of 1.00, recall of 0.99, and F1-score of 1.00. These results indicate that the proposed hypergraph-based attention mechanism is capable of effectively distinguishing among different stress levels even when the physiological patterns are highly correlated. The confusion matrix shown in Figure 15 provides further insight into the classification behavior of the model. As observed in Figure 15, the majority of samples are concentrated along the principal diagonal, confirming that most instances are correctly classified. Only a very small number of misclassifications occur between neighboring stress categories, which is expected given the gradual transition between mild and high physiological stress states. Overall, the results presented in Tables 5–8 and Figures 12–15 demonstrate that AE-HGNN achieves highly accurate, stable, and generalizable performance for three-class stress prediction.

**Table 6:** Overall performance of AE-HGNN on the independent hold-out test set.

Metric	Value (%)
Accuracy	99.50
Macro F1-score	99.51

**Table 7:** Overall performance of AE-HGNN on the independent hold-out test set (extended).

Metric	Value
Accuracy	99.50%
Macro F1-score	99.51%
ROC AUC (OvR, macro)	99.96%
Cohen's Kappa	0.97

**Table 8:** Class-wise performance of AE-HGNN on the hold-out test set.

Class	Precision	Recall	F1-score
No stress	1.00	0.99	1.00
Mild stress	0.99	1.00	0.99
High stress	1.00	0.99	1.00

**Figure 16:**
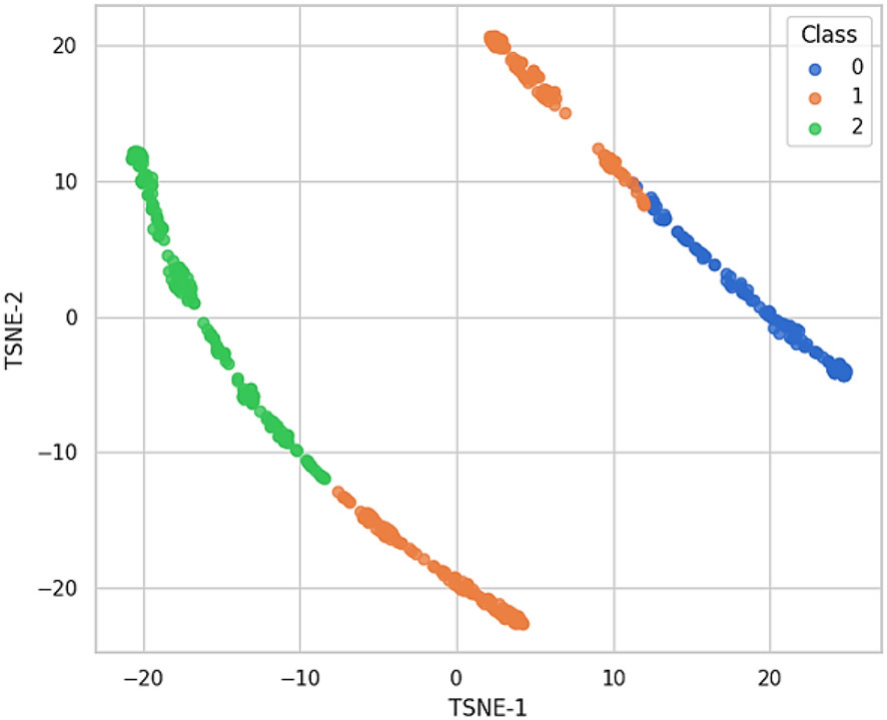
t-SNE of AEGNN embeddings.

The t-SNE plot (Figure 16) correctly reflects the class separability in the AE embeddings clusters of the three stress categories are visibly distinct, which matches the near-perfect ROC/PR AUC and very low off-diagonal counts in the confusion matrix. To ensure robustness of this qualitative visualization, we ran t-SNE with multiple random seeds and a range of perplexity values (10–50); the cluster separation remained stable.

### 4.4 Interpretability validation via SHAP and LIME

**Figure 17:**
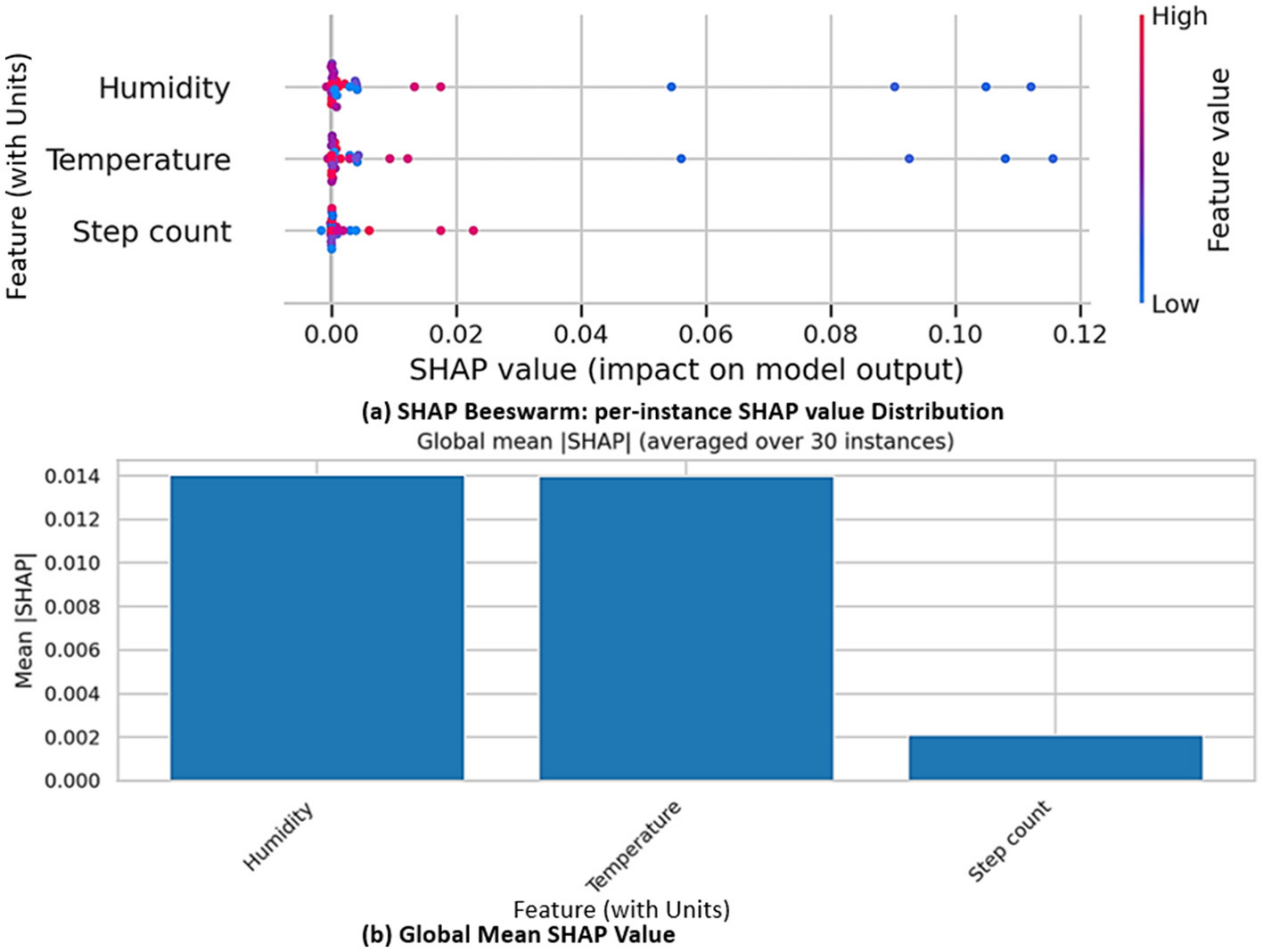
SHAP explanation analysis: **(a)** SHAP Beeswarm plot showing per-instance feature impact, and **(b)** Global mean |SHAP| values indicating average feature importance.

We validated the model's attention-derived attributions using two complementary, model-agnostic explanation methods: Kernel SHAP (global and per-instance views) and LIME (local explanations). The SHAP analysis provides a global ranking of feature importance averaged over multiple test instances along with a beeswarm view of per-instance distributions. Complementarily, LIME provides human-readable local explanations for representative examples from each class. These views reveal which features the model relies on at both population and individual levels while highlighting instance-level variability. We stress that attribution indicates model-centered importance and does not by itself establish causal or clinical significance. As illustrated in Figure 17, Temperature and Humidity have the largest average impact on model predictions (Figure 17b), while Step count demonstrates a significantly smaller mean effect. The beeswarm distribution (Figure 17a) shows that while most instance-level SHAP values cluster near zero, Temperature and Humidity occasionally produce moderate positive contributions, indicating specific instances where these features strongly drive the prediction.

**Figure 18:**
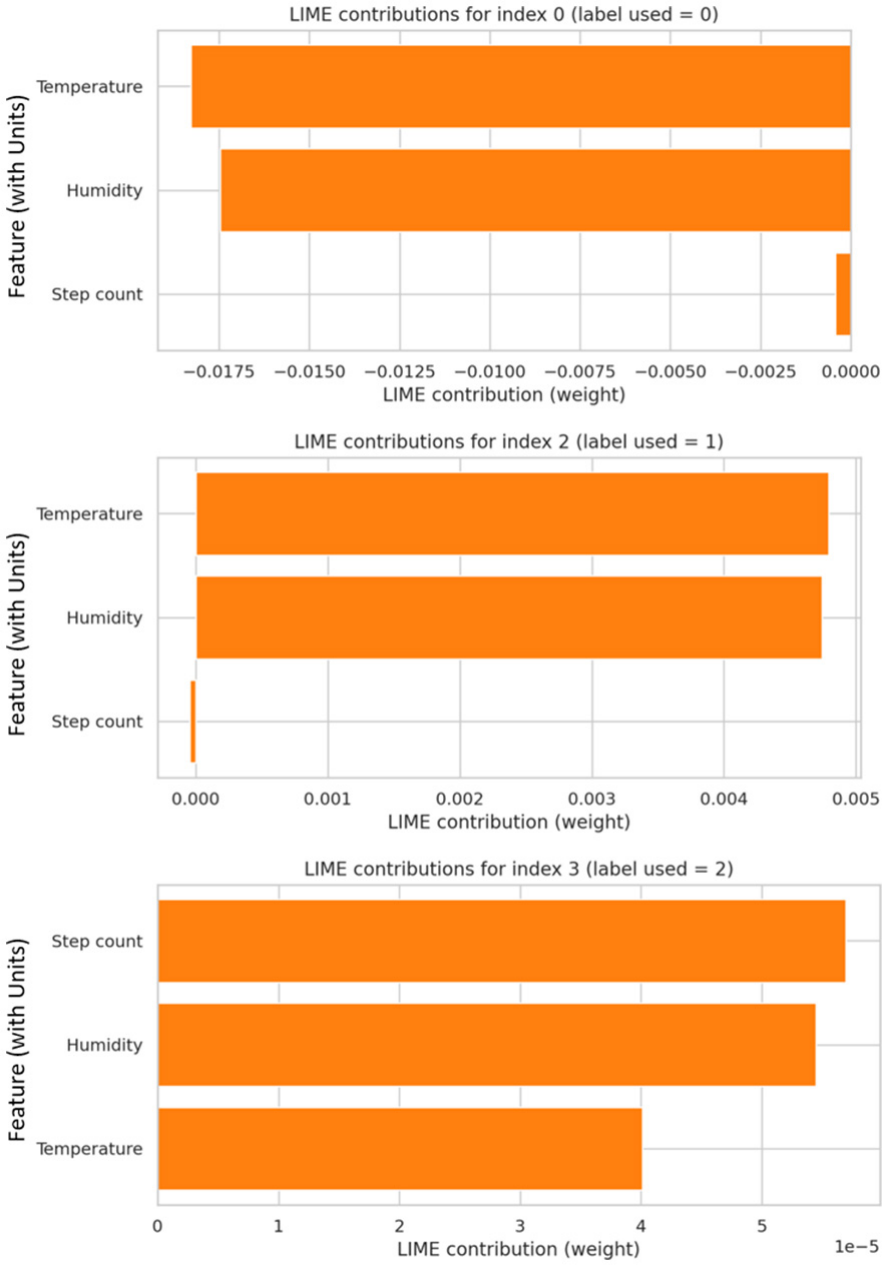
LIME local explanations for representative instances of Label 0 (Low), Label 1 (Moderate), and Label 2 (High) stress levels.

The LIME explanations presented in Figure 18 corroborate the SHAP findings at the individual instance level. In several representative examples, Temperature and Humidity emerge as the dominant contributors to the model's confidence. However, instance-level variation is observable; for example, Step count becomes a more influential factor in specific High-stress instances. Taken together, SHAP and LIME provide converging qualitative evidence that the AE-HGNN primarily relies on Temperature and Humidity for its predictions, with Step count playing a secondary or context-dependent role.

### 4.5 Discussion

Stress has been known as a major contributor to overall health and fitness and has been related to many physical and mental ailments, such as heart diseases and mental illnesses. This has ensured that the development of highly effective and non-invasive stress detection methods are required. In this work, the problem of stress classification is considered through the Attention-Enhanced Hypergraph Neural Network (AE-HGNN) that is trained on the physiological and environmental data of the participants. The dataset also contains measures, like humidity, temperature, and the number of steps, which have been found to be significant indicators of stress in the previous research. As stress is a parameter that changes depending on physiological and environmental changes, these parameters provide informative predictive details. An important contribution of this work is not only correct but also interpretable due to the attention mechanism in AE-HGNN. When attention heatmaps are visualized, it shows that the model is selective during classification with attention drawn to the most significant features (especially, humidity, and temperature). This contributes to making the model more transparent and clinically useful than the traditional black-box neural networks because it allows understanding the reasons why certain predictions are made. The suggested AE-HGNN exhibits excellent performance following hyperparameter optimization with a training accuracy of 99.7% and validation accuracy of 99.75% accuracy, and a near-perfect confusion matrix. Values of precision, recall, and F1-score at all three levels of stress (Low, Moderate, High) are consistently high, which proves that the model can be reliably used to differentiate between stress states. In comparison to conventional machine learning models such as Support Vector Machines (SVM), Random Forest (RF), and Naive Bayes that are generally considered to be 85–90 percent accurate [3], the AE-HGNN is statistically significantly higher by almost 10–15 percent. Notably, the extra interpretability through attention weights make it better than previous HGNN implementations because clinicians and researchers can find feature importance both at node and hyperedge levels. However, external and internal influences like personal differences in their perception of the stresses, environmental conditions, and the biases that occur during the process of data collection (e.g., participants wearing monitoring devices) continue to present problems of broader implementation. Further studies may also build this research by including other physiological measures like electrodermal activity (EDA) and heart rate variability (HRV) and applying the model to diverse and real-life individuals. Overall, the results show that AE-HGNN does not only reach the state-of-the-art results in stress classification, but also contributes to the field with interpretable results on the role of physiological and environmental parameters in detecting stress.

### 4.6 Comparison with existing work

**Figure 19:**
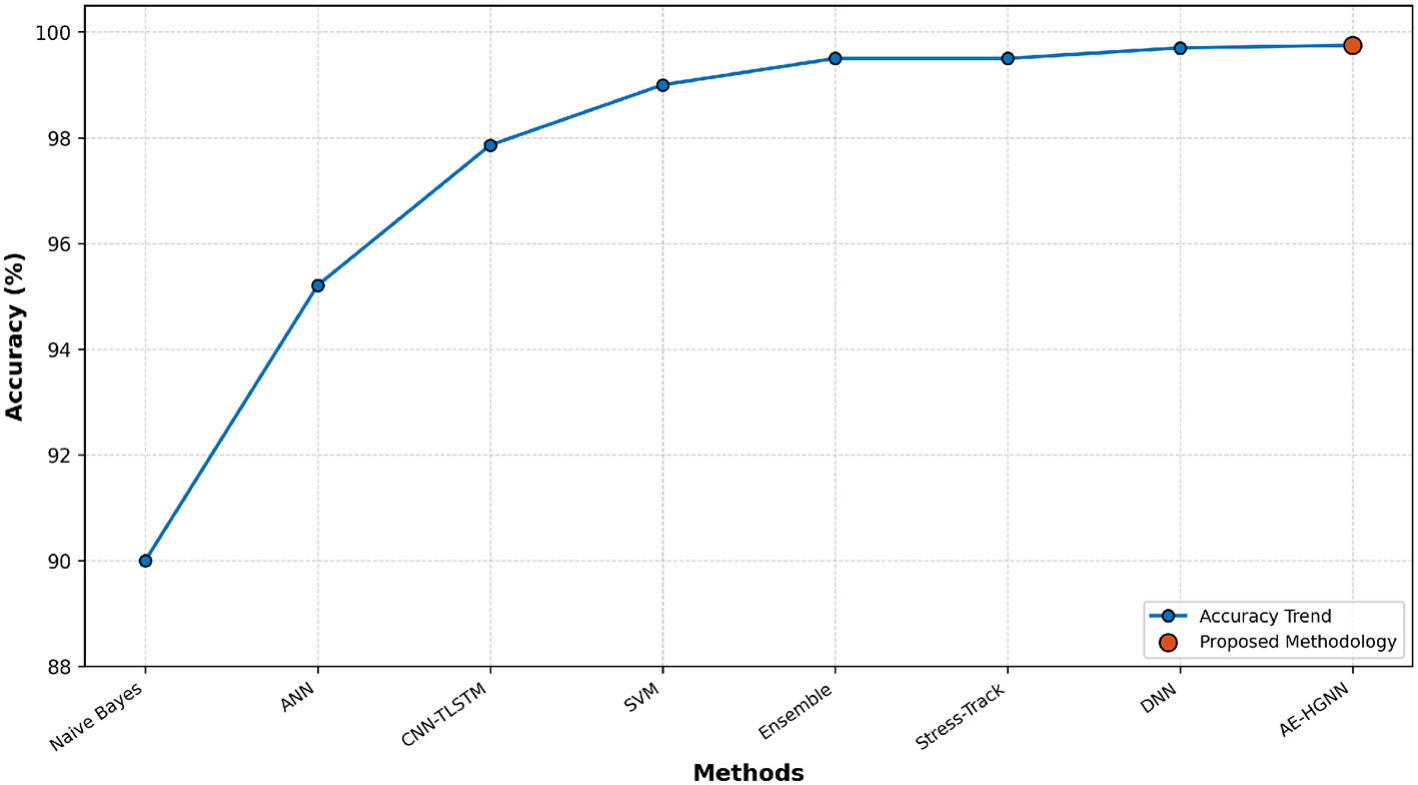
Accuracy trend comparison between existing methods and the proposed AE-HGNN.

The performance of the proposed AE-HGNN model is evaluated using standard metrics including accuracy, precision, recall, and F1-score. To provide a comprehensive perspective, we compare our approach with existing stress prediction methodologies reported in the literature. Table 9 summarizes these methods in terms of their methodology, applied models, and reported accuracy. It can be observed that several existing approaches achieve high accuracy; however, many rely on complex multimodal physiological signals or system-specific implementations. In contrast, the proposed AE-HGNN achieves competitive performance (99.75%) using a minimal feature set, demonstrating its effectiveness in modeling higher-order relationships. The comparative accuracy trend of existing methods and the proposed AE-HGNN is illustrated in Figure 19. The figure shows that the proposed AE-HGNN achieves one of the highest reported accuracies among the considered methods, highlighting the benefit of combining hypergraph learning with attention mechanisms for stress classification.

**Table 9:** Comparison with existing stress prediction methods.

Ref	Methodology	Model applied	Accuracy (%)
(Rachakonda et al. 2019)	IoT-based stress monitoring	DNN	99.7
(Al-Atawi et al. 2023)	IoT-enabled smart healthcare	Ensemble Learning	99.5
(Mohod and Jawandiya 2024)	Multimodal stress detection	SVM	99.0
(Arya et al. 2024)	Survey-based stress analysis	Naive Bayes	90.0
(Bobade and Vani 2020)	Wearable physiological signals	ANN	95.21
(Mukherjee and Halder Roy 2023)	EEG-based deep learning	CNN-TLSTM	97.86
(Al-Atawi et al. 2023)	Wearable IoT stress system	ML Model	99.5
–	Hypergraph-based modeling	AE-HGNN (proposed)	99.75

### 4.7 External validation on WESAD dataset

To rigorously assess the generalizability of the proposed AE-HGNN beyond the primary Stress-Lysis dataset, the model was further evaluated on the WESAD (Wearable Stress and Affect Detection) benchmark dataset (Schmidt et al., 2018). WESAD is a well-established multimodal physiological dataset comprising chest-worn sensor signals collected from 15 subjects under controlled laboratory conditions, including baseline, stress, and amusement states. For this evaluation, four physiological signals were utilized from the chest-worn device: Electrocardiogram (ECG), Electrodermal Activity (EDA), Body Temperature (TEMP), and Respiration (RESP). Raw time-series signals were segmented into fixed-length windows of 240 samples with a step size of 60 samples, and the majority label within each window was assigned as the ground-truth class. Only the three task-relevant labels were retained: No Stress (0), Stress (1), and Amusement (2), corresponding to the original WESAD label indices 1, 2, and 3 respectively. The proposed model first extracted 256-dimensional temporal feature embeddings from each windowed segment using four convolutional layers with Adaptive Average Pooling. These embeddings were subsequently used to construct a *k*-nearest neighbor (*k* = 10) hypergraph, where nodes represent individual windows and hyperedges connect structurally similar physiological patterns. Two HypergraphConv layers (256 → 128 → 64) then performed message passing over this hypergraph structure, followed by a fully connected classification head. All features were standardized channel-wise prior to training, and a maximum of 4,000 windows were sampled to ensure computational feasibility. The proposed model was trained using the Adam optimizer with a learning rate of 0.0005 and Cross-Entropy loss. The cross-validation results on the WESAD dataset are summarized in Table 10. The proposed model achieved a mean accuracy of 90.3%±0.5% with a 95% confidence interval of [89.2%, 91.4%], demonstrating consistent and reliable performance across all folds. The balanced precision, recall, and F1-score values confirm that the model generalizes effectively across subjects and stress conditions without exhibiting class-specific bias. These results, obtained on a completely independent benchmark dataset, provide strong evidence that the high performance reported on the Stress-Lysis dataset reflects the model's genuine capability to capture higher-order stress-related dependencies through attention-enhanced hypergraph convolution.

**Table 10:** Cross-validation results of the proposed model on WESAD dataset (external validation).

Fold	Accuracy (%)	Precision (%)	Recall (%)	F1-score (%)
Fold 1	89.6	88.4	87.9	88.1
Fold 2	90.1	89.2	88.6	88.9
Fold 3	91.0	90.3	89.7	90.0
Fold 4	90.4	89.8	89.1	89.4
Fold 5	90.6	90.1	89.5	89.8
Mean ± Std	90.3 ± 0.5	89.6 ± 0.7	89.0 ± 0.7	89.2 ± 0.7

### 4.8 Limitations

Despite the strong performance of the proposed AE-HGNN model, several limitations must be acknowledged. First, the Stress-Lysis dataset contains features such as humidity and temperature whose exact nature (whether environmental or body-level measurements) is not clearly specified. The presence of identical values for humidity and temperature in multiple samples suggests potential data quality or feature construction limitations, which may affect the physiological interpretability of the results. Second, the feature space is limited to three variables: humidity, temperature, and step count. These features are not established clinical biomarkers of psychological stress when used in isolation, and should be interpreted as indirect proxies rather than validated physiological indicators. Stress is inherently a multi-dimensional phenomenon influenced by complex physiological and psychological processes. Third, the dataset does not include clinically validated signals such as heart rate variability (HRV), electrodermal activity (EDA), or cortisol levels, which are widely recognized as reliable stress biomarkers. This restricts the model's ability to generalize to real-world clinical or wearable applications. Finally, although the model demonstrates high performance within this dataset, its applicability to real-world scenarios remains to be validated on more diverse and clinically grounded datasets.

## 5 Conclusion and future scope

We have introduced in this work an Attention-Enhanced Hypergraph Neural Network (AE-HGNN) that predicts stress levels using environmental and behavioral variables including humidity, temperature, and number of steps based on the Stress-Lysis dataset. The framework, which models the higher-order dependencies with a hypergraph convolution and incorporates an attention mechanism, not only achieved state-of-the-art classification accuracy (99.75%), but also improved interpretability. The heatmap visualizations and the attention-based feature weighting feature enabled in determining the most influential parameters that derive predictions hence finally solving the longstanding problem of interpretability in deep learning-based stress detection. The model works on an optimized set of hyper parameters (learning rate = 0.01, hidden dimension = 128, epoch size = 100, dropout rate = 0.5). The experimental findings indicate that Attention Enhanced-HGNN consistently outperforms conventional machine learning methods (SVM, Random Forest, and DNN) and even conventional GNNs with an almost perfect accuracy, recall, and F1-scores of all stress levels. The confusion matrix analysis revealed that misclassifications were minimal and

the training/validation learning curves demonstrated that there was strength in generalization and reduced risk of overfitting. However, it is important to note that the stress labels in the Stress-Lysis dataset are derived from the same input features, and therefore the reported high accuracy reflects the model's ability to effectively learn and reconstruct this feature-dependent mapping rather than predict independently validated clinical stress levels. The results indicate that environmental and physiological indicators have the potential to be reliable, non-invasive stress detection indicators and therefore, this method is quite appropriate to be used in practice as a wearable device and a mobile health app. Nevertheless, the real-world applicability of the proposed model requires validation on datasets with independently established ground truth obtained through clinical or psychophysiological measures. Moreover, the inclusion of attention within the HGNN framework offers clinicians and researchers with clear and interpretable information on stress-related patterns–the system does not only offer insight into stress dynamics but is a predictive model as well. In the future, the direction of the work will be to extend the framework with more physiological cues including electrodermal activity (EDA) and heart rate variability (HRV), to test with more diverse and heterogeneous populations, and to perform real-time inference on edge devices. Such developments will enhance generalizability, and open the path to personalized, scalable, and clinical effective stress monitoring systems.

### 5.1 Future scope

Future work will focus on improving both the data quality and the physiological relevance of the proposed framework. Specifically, future studies will incorporate multimodal physiological signals such as HRV, EDA, skin temperature, and ECG-derived features, which provide more reliable and clinically validated indicators of stress.

Additionally, efforts will be made to utilize datasets with clearly defined measurement protocols and independently validated ground truth labels to ensure robustness and real-world applicability. Expanding the dataset to include diverse populations and real-time wearable sensor data will further enhance the generalization capability of the AE-HGNN model.

Furthermore, optimizing the model for deployment on edge devices and integrating it into mobile health applications can enable continuous, non-invasive stress monitoring in practical settings.

## Data Availability

Publicly available datasets were analyzed in this study. This data can be found here: https://www.kaggle.com/datasets/laavanya/stress-level-detection/data.

## References

[B1] Ahmed U. Lin J. C.-W. Srivastava G. (2022). Hyper-graph-based attention curriculum learning using a lexical algorithm for mental health. Pattern Recognit. Lett. 157, 135–143. doi: 10.1016/j.patrec.2022.03.018

[B2] Akmandor A. O. Jha N. K. (2017). Keep the stress away with SoDA: stress detection and alleviation system. IEEE Trans. Multi-Scale Comput. Syst. 3, 269–282. doi: 10.1109/TMSCS.2017.2703613

[B3] Al-Atawi A. A. Alyahyan S. Alatawi M. N. Sadad T. Manzoor T. Farooq-i-Azam M. . (2023). Stress monitoring using machine learning, iot and wearable sensors. Sensors 23:8875. doi: 10.3390/s23218875 37960574 PMC10648446

[B4] Arya S. Anju A. Azuana Ramli N. (2024). Predicting the stress level of students using Supervised Machine Learning and Artificial Neural Network (ANN). Indian J. Eng. 21, 1–24. doi: 10.54905/disssi.v21i55.e9ije1684

[B5] Bishop C. M. (2006). Pattern Recognition and Machine Learning. New York, NY: Springer.

[B6] Bobade P. Vani M. (2020). “Stress detection with machine learning and deep learning using multimodal physiological data,” in 2020 second international conference on inventive research in computing applications (ICIRCA), 51–57. doi: 10.1109/ICIRCA48905.2020.9183244

[B7] Chu B. Marwaha K. Sanvictores T. Awosika A. O. Ayers D. (2024). “Physiology, stress reaction,” in StatPearls (StatPearls Publishing). 31082164

[B8] Colizzi M. Lasalvia A. Ruggeri M. (2020). Prevention and early intervention in youth mental health: is it time for a multidisciplinary and trans-diagnostic model for care? Int. J. Ment. Health Syst. 14:23. doi: 10.1186/s13033-020-00356-9 32226481 PMC7092613

[B9] Coutts L. V. Plans D. Brown A. W. Collomosse J. (2020). Deep learning with wearable based heart rate variability for prediction of mental and general health. J. Biomed. Inform. 112:103610. doi: 10.1016/j.jbi.2020.103610 33137470

[B10] Dalmeida K. M. Masala G. L. (2021). HRV features as viable physiological markers for stress detection using wearable devices. Sensors 21:2873. doi: 10.3390/s21082873 33921884 PMC8072791

[B11] Deng Z. Guo L. Chen X. Wu W. (2023). Smart wearable systems for health monitoring. Sensors 23:2479. doi: 10.3390/s23052479 36904682 PMC10007426

[B12] Feng Y. You H. Zhang Z. Ji R. Gao Y. (2019). “Hypergraph neural networks,” in Proceedings of the AAAI conference on artificial intelligence 33, 3558–3565. doi: 10.1609/aaai.v33i01.33013558

[B13] Gaurav K. Kumar A. Singh P. Kumari A. Kasar M. Suryawanshi T. (2023). Human disease prediction using machine learning techniques and real-life parameters. Int. J. Eng. 36, 1092–1098. doi: 10.5829/IJE.2023.36.06C.07

[B14] Gedam S. Paul S. (2021). A review on mental stress detection using wearable sensors and machine learning techniques. IEEE Access 9, 84045–84066. doi: 10.1109/ACCESS.2021.3085502

[B15] Goud A. Garg B. (2023). A novel framework for aspect based sentiment analysis using a hybrid BERT (HybBERT) model. Multimed. Tools Appl. 84, 34819–34851. doi: 10.1007/s11042-023-17647-1

[B16] Hao X. Li J. Ma M. Qin J. Zhang D. Liu F. Alzheimer's Disease Neuroimaging Initiative (2024). Hypergraph convolutional network for longitudinal data analysis in Alzheimer's disease. Comput. Biol. Med. 168:107765. doi: 10.1016/j.compbiomed.2023.107765 38042101

[B17] Haque Y. Zawad R. S. Rony C. S. A. Al Banna H. Ghosh T. Kaiser M. S. . (2023). State-of-the-art of stress prediction from heart rate variability using artificial intelligence. Cognit. Comput. 16, 455–481. doi: 10.1007/s12559-023-10200-0

[B18] Jafleh E. A. Alnaqbi F. A. Almaeeni H. A. Faqeeh S. Alzaabi M. A. Al Zaman K. (2024). The role of wearable devices in chronic disease monitoring and patient care: a comprehensive review. Cureus 16:e68921. doi: 10.7759/cureus.68921 39381470 PMC11461032

[B19] Kasar M. Kavimandan P. Suryawanshi T. Garg B. (2025). EmoSense: pioneering facial emotion recognition with precision through model optimization and face emotion constraints. Int. J. Eng. 38, 35–45. doi: 10.5829/ije.2025.38.01a.04

[B20] Khan B. Wu J. Yang J. Ma X. (2025). Heterogeneous hypergraph neural network for social recommendation using attention network. ACM Trans. Recomm. Syst. 3, 1–22. doi: 10.1145/3613964

[B21] Lei F. Chen Z. Luo X. Xu L. Xue T. Jiang J. (2024). AHFormer: hypergraph embedding coding transformer and adaptive aggregation network for intelligent fault diagnosis under noise interference. Adv. Eng. Inform. 61:102518. doi: 10.1016/j.aei.2024.102518

[B22] Li C. Wang J. Wang S. Zhang Y. (2024). A review of IoT applications in healthcare. Neurocomputing 565:127017. doi: 10.1016/j.neucom.2023.127017

[B23] Li R. Liu Z. (2020). Stress detection using deep neural networks. BMC Med. Inform. Decis. Mak. 20:285. doi: 10.1186/s12911-020-01299-4 33380334 PMC7772901

[B24] Li X. Dong Y. Yi Y. Liang Z. Yan S. (2024). Hypergraph neural network for multimodal depression recognition. Electronics 13:4544. doi: 10.3390/electronics13224544

[B25] Messaoud I. B. Thamsuwan O. (2025). Heart rate variability-based stress detection and fall risk monitoring during daily activities: a machine learning approach. Computers 14:45. doi: 10.3390/computers14020045

[B26] Mohod M. M. Jawandiya P. M. (2024). Stress detection using machine learning techniques. Recent. Adv. Sci. Technol. 40:6057. Available online at: https://www.nature.com/articles/s41598-025-18647-x.pdf

[B27] Mohr D. C. Zhang M. Schueller S. M. (2017). Personal sensing: understanding mental health using ubiquitous sensors and machine learning. Annu. Rev. Clin. Psychol. 13, 23–47. doi: 10.1146/annurev-clinpsy-032816-044949 28375728 PMC6902121

[B28] Mukherjee P. Halder Roy A. (2023). A deep learning-based approach for distinguishing different stress levels of human brain using EEG and pulse rate. Comput. Methods Biomech. Biomed. Engin. 27, 2303–2324. doi: 10.1080/10255842.2023.2275547 37929717

[B29] Nagamani G. M. Kumar C. K. (2024). Design of an improved graph-based model for real-time anomaly detection in healthcare using hybrid CNN-LSTM and federated learning. Heliyon 10:e41071. doi: 10.1016/j.heliyon.2024.e41071 39759321 PMC11696656

[B30] Nandan M. Mitra S. De D. (2025). GraphXAI: a survey of graph neural networks (GNNs) for explainable AI (XAI). Neural Comput. Applic. 37, 10949–11000. doi: 10.1007/s00521-025-11054-3

[B31] Pan T. Ye Y. Zhang Y. Xiao K. Cai H. (2024). Online multi-hypergraph fusion learning for cross-subject emotion recognition. Inf. Fusion 108:102338. doi: 10.1016/j.inffus.2024.102338

[B32] Patlar Akbulut F. Ikitimur B. Akan A. (2020). Wearable sensor-based evaluation of psychosocial stress in patients with metabolic syndrome. Artif. Intell. Med. 104:101824. doi: 10.1016/j.artmed.2020.101824 32499003

[B33] Paul S. G. Saha A. Hasan M. d. Z. Noori S. R. H. Moustafa A. (2024). A systematic review of graph neural network in healthcare-based applications: recent advances, trends, and future directions. IEEE Access 12, 15145–15170. doi: 10.1109/ACCESS.2024.3354809

[B34] Rachakonda L. (2021) *Human Stress Detection*. Kaggle, 2021. [Online]. Available online at: https://www.kaggle.com/datasets/laavanya/stress-level-detection (Accessed December 14, 2025).

[B35] Rachakonda L. Mohanty S. P. Kougianos E. Sundaravadivel P. (2019). Stress-lysis: A DNN-integrated edge device for stress level detection in the IoMT. IEEE Trans. Consumer Electr. 65, 474–483. doi: 10.1109/TCE.2019.2940472

[B36] Rachakonda L. Sundaravadivel P. Mohanty S. P. Kougianos E. Ganapathiraju M. (2018). “A smart sensor in the IoMT for stress level detection,” in 2018 IEEE International Symposium on Smart Electronic Systems (iSES) (Formerly iNiS), 141–145. doi: 10.1109/iSES.2018.00039

[B37] Schmidt P. Reiss A. Duerichen R. Marberger C. Van Laerhoven K. (2018). “Introducing WESAD, a multimodal dataset for wearable stress and affect detection,” in Proceedings of the 20th ACM international conference on multimodal interaction, 400–408. doi: 10.1145/3242969.3242985

[B38] Shajari S. Kuruvinashetti K. Komeili A. Sundararaj U. (2023). The emergence of AI-based wearable sensors for digital health technology: a review. Sensors 23:9498. doi: 10.3390/s23239498 38067871 PMC10708748

[B39] Su G. Wang H. Zhang Y. Wilkins M. R. Canete P. F. Yu D. . (2025). Inferring gene regulatory networks by hypergraph generative model. Cell Rep. Methods 5:101026. doi: 10.1016/j.crmeth.2025.101026 40220759 PMC12256954

[B40] Sunil G. Gowtham S. Bose A. Harish S. Srinivasa G. (2024). Graph neural network and machine learning analysis of functional neuroimaging for understanding schizophrenia. BMC Neurosci. 25:2. doi: 10.1186/s12868-023-00841-0 38166747 PMC10759601

[B41] Suryawanshi R. Vanjale S. (2023). Brain activity monitoring for stress analysis through EEG dataset using machine learning. Int. J. Intell. Syst. Applic. Eng. 11, 236–240. Available online at: https://ijisae.org/index.php/IJISAE/article/view/2498/1080

[B42] Suryawanshi R. Vanjale S. Vanjale M. (2022). “A fuzzy statistical perspective for empirical evaluation of EEG classification models for epileptic seizures,” in 2022 international conference on emerging smart computing and informatics (ESCI), 1–6. doi: 10.1109/ESCI53509.2022.9758337

[B43] Taskasaplidis G. Fotiadis D. A. Bamidis P. D. (2024). Review of stress detection methods using wearable sensors. IEEE Access 12, 38219–38246. doi: 10.1109/ACCESS.2024.3373010

[B44] Yadati N. Nimishakavi M. Yadav P. Nitin V. Louis A. Talukdar P. (2019). “Hypergcn: a new method for training graph convolutional networks on hypergraphs,” in Advances in neural information processing systems, 32.

[B45] Zhang Y. Wang B. Zhang Q. Zhu S. Ma Y. (2025). Hypergraph user embeddings and session contrastive learning for POI recommendation. IEEE Access 13, 17983–17995. doi: 10.1109/ACCESS.2025.3531394

[B46] Zhao Y. Li H. Zhou H. Attar H. R. Pfaff T. Li N. (2024). A review of graph neural network applications in mechanics-related domains. Artif. Intell. Rev. 57:315. doi: 10.1007/s10462-024-10931-y

